# Mechanisms Underlying the Coordination of EMT and Glycolysis Mediated by Hypoxia and Glucose

**DOI:** 10.3390/cimb48090907

**Published:** 2026-09-04

**Authors:** Wei Lu, Hang-Yu Wang, Xiao-Peng Zhang, Wei Wang

**Affiliations:** 1 Kuang Yaming Honors School, Nanjing University, Nanjing 210023, China; 2 National Laboratory of Solid State Microstructure, Department of Physics, Nanjing University, Nanjing 210093, China; 3 Institute for Brain Sciences, Nanjing University, Nanjing 210023, China

**Keywords:** epithelial–mesenchymal transition, glycolysis, hypoxia, glucose availability, bistability, hysteresis, phenotypic memory, cancer metabolism

## Abstract

Hypoxia is a hallmark of the tumor microenvironment. Under hypoxia, HIF-1α accumulates and promotes both epithelial–mesenchymal transition (EMT) and glycolysis depending on glucose levels. However, how EMT and glycolysis are coordinated by oxygen and glucose abundance is still not well understood. Here, we developed an integrated model to investigate the mechanism underlying the regulation of EMT and glycolysis at varying oxygen and glucose levels. We focused on how the interplay between EMT and glycolysis maintains cell phenotypes. Our results show that hypoxia and sufficient glucose facilitate the transition of cells toward an invasion-associated mesenchymal–glycolytic phenotype. Moreover, enhanced glycolysis promotes the completion of EMT and reinforces the intermediate states. Under glucose-sufficient conditions, the reciprocal promotion between EMT and glycolysis may convert transient hypoxia into persistent mesenchymal memory that maintains the mesenchymal phenotype after reoxygenation. Our work clarifies how metabolic microenvironmental fluctuations are transformed into durable invasion-associated phenotypic states. Our work may provide insights into therapies that target both the EMT and glycolysis pathways.

## 1. Introduction

Tumor cells are exposed to a series of microenvironmental factors, such as hypoxia, inflammation, nutritional deficiency, acidosis, and oxidative stress [1]. These external cues are converted into phenotypic outcomes of tumor cells such as metabolic reprogramming, metastasis, survival, and death through various cell signaling pathways [2,3]. It is challenging to explore how different signaling pathways interact in the fluctuating tumor microenvironment.

As a critical step in metastasis, epithelial–mesenchymal transition (EMT) exhibits decreased expression of epithelial markers (such as E-cadherin, occludin, and claudin) and increased expression of mesenchymal markers (such as N-cadherin, vimentin, and fibronectin) [4]. EMT can be induced by several signals, including transforming growth factor-beta (TGF-β), hepatocyte growth factor (HGF), and hypoxia. It is regulated by EMT-inducing transcription factors (EMT-TFs) such as SNAIL, ZEB, and TWIST, which repress E-cadherin expression [4]. TGF-β-mediated EMT has been intensively investigated, and the SNAIL-miR-34a and ZEB-miR-200 positive feedback loops have been identified as the core module for inducing EMT [4]. As a typical microenvironmental property in solid tumors, hypoxia can promote EMT through hypoxia-inducible factor-1α (HIF-1α), which induces the production of EMT-TFs [5]. However, the mechanism of hypoxia-dependent pathways in EMT regulation still needs further investigation.

Under hypoxia, metabolic reprogramming often accompanies EMT: the mode of energy metabolism usually switches from oxidative phosphorylation (OXPHOS) to glycolysis [6]. In this case, cells produce ATP rapidly but inefficiently, with lactate as a byproduct [7]. When oxygen is deficient, HIF-1α promotes glycolysis by transactivating genes including *GLUT1*, *HK2*, *PFK1*, and *LDHA*, whose products are involved in different key steps of glycolysis [8]. Moreover, cancer cells largely depend on glycolysis for energy production even in the presence of sufficient oxygen, a phenomenon referred to as the Warburg effect or aerobic glycolysis [9]. Aerobic glycolysis is an aggressive metabolic phenotype that provides sufficient ATP to support the rapid proliferation of tumor cells [10]. These cells must take up substantially more glucose than normal cells [7]. Thus, glucose abundance is also a key factor in the regulation of glycolysis, controlling glycolytic flux and lactate accumulation. It is intriguing to explore how hypoxia and glucose availability jointly regulate glycolysis.

Experimental studies have revealed a bidirectional, mutually reinforcing interplay between EMT and glycolysis [3,11,12]. On one hand, EMT-TFs can enhance glycolysis by upregulating enzymes such as lactate dehydrogenase A (LDHA) and promoting the expression of glucose transporters like GLUT1, thereby increasing glucose uptake and lactate production [11,12]. On the other hand, lactate can stabilize HIF-1α and facilitate EMT [13,14]. Together, these findings suggest that EMT and glycolysis mutually promote each other under hypoxia [3,12]. It is necessary to investigate how the interplay between EMT and glycolysis modulates the stability of each cellular outcome.

Due to the complexity of the regulation of EMT and glycolysis, a series of models have been developed to reveal the mechanism underlying their regulation by various signals. Tian et al. proposed that two interlinked positive feedback loops contribute to the reversible and irreversible switches during EMT [15]. Lu et al. proposed that SNAIL and ZEB play different roles in the modulation of EMT [16]. Wang et al. investigated the link between the number of positive feedback loops in the HIF-1α network and the number of stable states in EMT under hypoxia [17]. We have previously clarified how cells make a decision between glycolysis and necrosis in response to hypoxia [18]. Several modeling studies have also focused on the dynamic mechanisms of glycolysis regulation [19,20]. Although most models have focused on the mechanism underlying either EMT or glycolysis, it is crucial to construct a comprehensive model to clarify how the two processes are coordinated in response to hypoxia and varying glucose levels.

Based on the above considerations, we developed an integrated model to characterize the coordination between EMT and glycolysis at varying oxygen and glucose levels. By combining numerical simulation, bifurcation analysis, and dynamic analysis, we show that hypoxia and sufficient glucose jointly drive cells toward a mesenchymal–glycolytic state. Moreover, glycolysis is predicted to function not only as a downstream output of EMT but also as a feedback stabilizer that expands the mesenchymal stability window, reinforces hybrid phenotypes, and converts transient hypoxia into stable mesenchymal memory. These insights provide a dynamical basis for understanding how microenvironmental fluctuations are transformed into persistent invasion-associated phenotypes.

## 2. Materials and Methods

**Figure 1:**
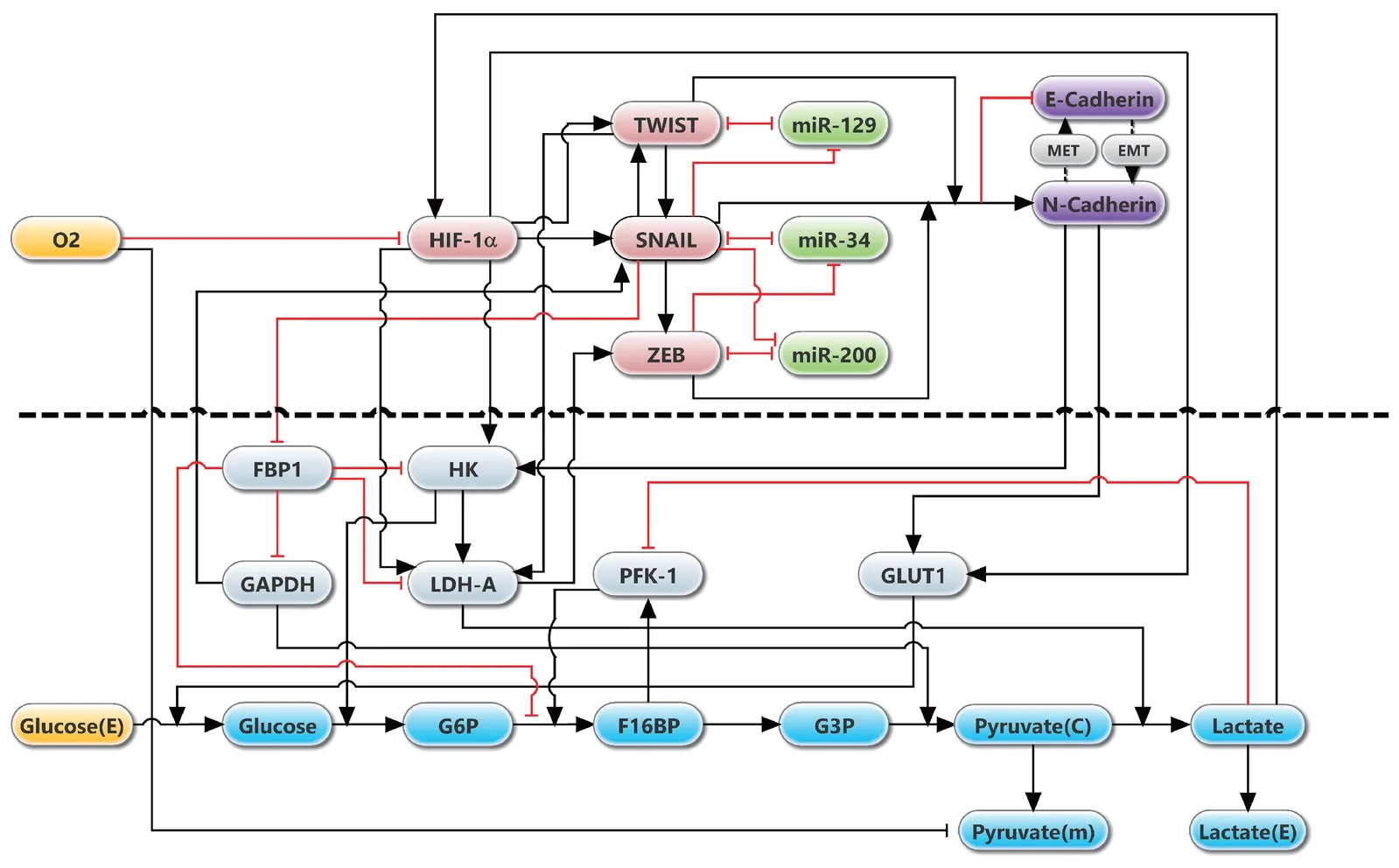
Schematic of the integrated EMT–glycolysis network model. The model couples the core EMT transcriptional network, driven by HIF-1α and governed by three miRNA–TF feedback loops, with the central glycolytic pathway. Oxygen level $L_{O_{2}}$ and extracellular glucose concentration act as dual microenvironmental inputs. Bidirectional arrows represent the key hypothesis of this study: a self-reinforcing crosstalk between cellular phenotype and metabolism. Black arrows indicate activation or conversion, red blunt-ended lines indicate inhibition, and the dashed horizontal line separates the EMT regulatory module from the glycolytic module. Node colors denote different classes of components in the model.

We developed an integrated model of the HIF-1α network to investigate how EMT and glycolysis mutually reinforce each other (Figure 1). The model contains two interconnected modules: a glycolytic module representing glucose-dependent metabolic reprogramming and an EMT regulatory module representing oxygen-dependent phenotypic regulation. In this framework, glycolytic activation feeds back to the HIF-1α–EMT circuit through lactate-dependent stabilization of HIF-1α, whereas EMT activation modulates glycolysis through SNAIL-mediated repression of FBP1. Thus, the coupled network may generate a stable invasive state characterized by both enhanced glycolytic flux and mesenchymal traits. The details of model construction are presented as follows.

### 2.1. Regulation of EMT by HIF-1α-Centered Regulatory Circuit

In the upstream EMT regulatory module, oxygen levels are sensed by HIF-1α. Under normoxia, HIF-1α is hydroxylated by prolyl hydroxylases and degraded through the VHL-proteasome pathway, whereas hypoxia inhibits hydroxylation and promotes HIF-1α accumulation [21,22]. This oxygen-dependent control was represented in the HIF-1α equation, in which oxygen promotes HIF-1α degradation. Because lactate has been reported to stabilize HIF-1α, lactate was included in the same equation as a metabolic input that reduces effective HIF-1α degradation (see Equation (S1) in the Supplementary Materials (SM)).

Accumulated HIF-1α links hypoxic signaling to EMT regulation mainly through inducing EMT-TFs. HIF-1α directly binds hypoxia-response elements to promote the transcription of *twist* and *snail* under hypoxic conditions [23,24]. Accordingly, HIF-1α was assumed to activate *snail* and *twist* transcription in the model (see Equations (S2) and (S6) in the Supplementary Materials). Because *twist* induction occurs earlier than *snail* induction in the EMT regulatory program, the Michaelis constant for HIF-1α-dependent *twist* production was set smaller than that for *snail* production [25]. Moreover, considering the upregulation of *zeb* transcription by SNAIL [26], the production rate of *zeb* mRNA was assumed to depend on the level of SNAIL in a Hill function (see Equation (S4) in the Supplementary Materials). Through these links, oxygen- and lactate-dependent HIF-1α regulation is transmitted to EMT-TFs and EMT marker dynamics.

The core circuit of EMT regulation is composed of EMT-TFs and epithelial-associated microRNAs. SNAIL, ZEB, and TWIST are major EMT-TFs that suppress epithelial programs and promote mesenchymal traits [27,28,29,30,31]. In the model, the mRNA variables *snail*, *zeb*, and *twist* were used to describe transcriptional regulation by upstream signals, and the corresponding protein variables SNAIL, ZEB, and TWIST were produced from these mRNAs (see Equations (S2)–(S7) in the Supplementary Materials).

These EMT-TFs are embedded in reciprocal inhibitory circuits with epithelial-associated microRNAs. The SNAIL/miR-34 and ZEB/miR-200 circuits were incorporated as canonical EMT decision-making motifs [32,33,34]. Mechanistically, miR-34 represses SNAIL mainly at the post-transcriptional level by targeting *snail* mRNA, whereas SNAIL represses miR-34 expression at the transcriptional level (see Equations (S2), (S3) and (S9) in the Supplementary Materials) [32]. Similarly, miR-200 represses ZEB mainly through post-transcriptional inhibition of *zeb* mRNA, whereas ZEB transcriptionally represses miR-200 expression (see Equations (S4), (S5) and (S10) in the Supplementary Materials) [33,34]. Under hypoxic conditions, the TWIST/miR-129 axis was also included, with miR-129 reducing TWIST protein production through post-transcriptional regulation and TWIST repressing miR-129 expression at the transcriptional level (see Equations (S7) and (S9) in the Supplementary Materials) [25]. Additional crosstalk among EMT regulatory loops was represented by transcriptional repression of miR-129 and miR-200 by SNAIL and repression of miR-34 by ZEB (see Equations (S8)–(S10) in the Supplementary Materials) [25,34,35,36]. Together, these interactions allow the EMT module to capture both post-transcriptional microRNA-mediated repression of EMT transcription factors and transcriptional repression of microRNAs by EMT transcription factors.

To read out EMT progression, epithelial and mesenchymal marker dynamics were included. E-cadherin (E-Cad) is a central epithelial adhesion molecule and is transcriptionally repressed by EMT-TFs such as SNAIL, ZEB, and TWIST [27,28,30,37]. Accordingly, E-Cad production was assumed to be inhibited by SNAIL, ZEB, and TWIST in the model (see Equation (S16) in the Supplementary Materials). N-cadherin (N-Cad) is a mesenchymal marker associated with the cadherin switch and increased motility during EMT [38]. Because EMT-TFs promote mesenchymal marker expression, N-Cad production was assumed to be induced by SNAIL, ZEB, and TWIST in the model (see Equation (S11) in the Supplementary Materials). Thus, E-Cad and N-Cad were used as epithelial and mesenchymal outputs of EMT progression, respectively.

### 2.2. Regulation of Glycolytic Metabolism

To focus on the interaction between glycolysis and HIF-1α–EMT regulation, we represented glycolysis as a reduced metabolic backbone rather than a complete reconstruction of all glycolytic intermediates [39]. The model retained the key processes required for this coupling, including glucose uptake, upper-glycolytic control, downstream glycolytic flux, pyruvate partitioning, and lactate production. Intermediate reactions not directly involved in the coupling mechanism were lumped into effective conversion steps, preserving the main functional structure of glycolysis while keeping the coupled model tractable [39,40].

In this reduced module, extracellular glucose (GLCE) is transported into the cell as intracellular glucose (GLC) through GLUT1. GLC is then phosphorylated by HK to form glucose-6-phosphate (G6P), committing glucose to intracellular metabolism [40,41]. The GLUT1- and HK-dependent terms were formulated as net reversible fluxes constrained by substrate/product saturation. The GLUT1 term describes bidirectional glucose transport between GLCE and GLC, whereas the HK term describes the net conversion between GLC and G6P. Accordingly, HK-mediated phosphorylation downregulates GLC and upregulates G6P (see Equations (S18) and (S19) in the Supplementary Materials).

Because fructose-6-phosphate (F6P) was not explicitly represented, the conversion from G6P to fructose-1,6-bisphosphate (F16BP) was modeled as a PFK-1-dependent effective upper-glycolytic step. This simplification preserves the major regulatory role of PFK-1 in upper glycolysis [42]. FBP1 was included as an anti-glycolytic regulator that counteracts the PFK-1-driven direction [43,44]. In the equations, this effective flux consumes G6P and produces F16BP and is modulated by G6P availability, PFK-1 activation, and FBP1 inhibition (see Equation (S19) in the Supplementary Materials).

F16BP was then converted into glyceraldehyde-3-phosphate (G3P), representing the lumped production of triose–phosphate intermediates without explicitly modeling DHAP [45,46]. This effective conversion decreases F16BP and increases G3P in the corresponding equations (see Equations (S21) and (S22) in the Supplementary Materials). G3P was further converted to cytosolic pyruvate (PYRc) through a GAPDH-associated downstream glycolytic flux. Because the intermediates between G3P and pyruvate were not explicitly modeled, this term represents a lumped downstream glycolytic segment rather than a single GAPDH-catalyzed reaction. Accordingly, the GAPDH-associated flux consumes G3P and produces PYRc (see Equations (S22) and (S23) in the Supplementary Materials) [39,45,46,47].

Cytosolic pyruvate (PYRc) was modeled as a downstream branching point. PYRc is produced by the GAPDH-mediated glycolytic flux and is then partitioned between LDHA-dependent lactate production and mitochondrial transport. LDHA catalyzes the reversible conversion between PYRc and lactate (LAC), supporting lactate production in glycolytic cancer cells [48]. The LDHA-dependent term was therefore written as a net reversible flux between PYRc and LAC; when the forward direction dominates, this flux consumes PYRc and produces LAC (see Equations (S23) and (S24) in the Supplementary Materials).

Finally, lactate exchange and mitochondrial pyruvate utilization were included to represent the two major downstream outcomes of pyruvate metabolism. Lactate shuttles between the intracellular and extracellular spaces, allowing the model to describe lactate accumulation and export (see Equations (S24) and (S25) in the Supplementary Materials). Alternatively, PYRc can be transported into the mitochondrial pyruvate pool (PYRm), where it is consumed through an oxidative utilization process [49]. This mitochondrial branch was used as a simplified representation of oxygen-dependent pyruvate utilization rather than a full reconstruction of acetyl-CoA production, the TCA cycle, and oxidative phosphorylation (see Equation (S26) in the Supplementary Materials). Together, these equations describe glucose uptake, glycolytic flux, pyruvate partitioning, and lactate accumulation in a reduced but mechanistically interpretable form.

### 2.3. Interplay Between Glycolysis and EMT

To couple the EMT module with the glycolytic metabolism module, we first introduced EMT-to-glycolysis regulatory links. HIF-1α was used as an oxygen-sensitive transcriptional regulator of glycolytic components. Under hypoxia, accumulated HIF-1α transcriptionally promotes genes involved in glucose uptake and glycolytic metabolism, including GLUT1, HK, and LDHA [50,51,52]. Accordingly, HIF-1α was assumed to activate GLUT1, HK, and LDHA expression in the model, linking oxygen-dependent signaling to glucose uptake, glucose phosphorylation, and pyruvate-to-lactate conversion (see Equations (S14), (S15) and (S17) in the Supplementary Materials).

EMT transcription factors were also connected to glycolytic regulation. TWIST-driven metabolic reprogramming has been reported to increase glucose consumption, lactate production, and LDHA expression in breast epithelial cells [53]. Thus, TWIST was considered an activator of LDHA production in the model (see Equation (S15) in the Supplementary Materials). SNAIL transcriptionally represses FBP1 to promote glycolysis in breast cancer cells [43]. This regulation was represented by SNAIL-dependent inhibition of FBP1 production (see Equation (S12) in the Supplementary Materials). Because FBP1 counteracts upper-glycolytic flux, reduced FBP1 relieves the anti-glycolytic constraint on the G6P-to-F16BP effective conversion (see Equations (S19) and (S21) in the Supplementary Materials).

N-Cad was included as a mesenchymal-state-associated input to GLUT1 and HK in the model. This link is a phenomenological connection used to capture the experimentally observed co-occurrence of mesenchymal marker expression with elevated glycolytic capacity under hypoxic or EMT-associated conditions [12,54,55]. Accordingly, N-Cad was assumed to promote GLUT1 and HK production as a proxy for mesenchymal-state-associated glycolytic activation (see Equations (S14) and (S17) in the Supplementary Materials). Together, these EMT-to-glycolysis links allow EMT-associated states to enhance glucose uptake, upper-glycolytic flux, and lactate production.

Conversely, the glycolysis module feeds back to the HIF-1α–EMT module through lactate and glycolysis-associated enzymes. Lactate is a major glycolytic product and has been reported to enhance HIF-1α protein stability and its transcriptional activity [13,56]. Therefore, intracellular lactate (LAC) was introduced as a metabolic feedback signal that reduces the effective degradation of HIF-1α (see Equation (S1) in the Supplementary Materials). Through this link, increased glycolytic flux and lactate accumulation can reinforce HIF-1α signaling.

Glycolytic enzymes were further included as regulators of EMT-TFs. GAPDH has been reported to enhance SNAIL expression in EMT [57]. This interaction was represented as GAPDH-dependent activation of *snail* production in the model (see Equation (S2) in the Supplementary Materials). LDHA was assumed to promote ZEB expression since LDHA knockdown suppresses ZEB2 and EMT-associated markers in cancer cells [58]. Because this promotional effect is indirect, the LDHA-to-ZEB link was modeled as a phenomenological activation of *zeb* expression (see Equation (S4) in the Supplementary Materials). Thus, glycolytic activity feeds back to EMT regulation through lactate-dependent HIF-1α stabilization and enzyme-associated activation of EMT-TFs.

### 2.4. Methods: Parameter Setting and Numerical Simulation

The details of model construction and parameter settings are provided in the Supplementary Materials. The integrated model was described by a set of ordinary differential equations (ODEs) to characterize the dynamics of all network components (see the Supplementary Materials, Section S1). The concentration of each component is represented by square brackets […]. Concentrations of regulatory molecular species, such as proteins, mRNAs, miRNAs, and intracellular metabolites, are expressed in μM, extracellular glucose concentration [GLCE] is expressed in mM, oxygen level is expressed as percentage O_2_, and time is expressed in hours.

For each network component, its synthesis was modeled as the sum of basal and regulated production, whereas degradation was modeled as a first-order process unless otherwise specified. Transcriptional or regulatory activation was represented by increasing Hill functions, whereas repression was represented by decreasing Hill functions. For glycolytic metabolites, the ODEs were formulated according to mass-balance principles, in which the rate of change of each metabolite equals the upstream production or transport flux minus downstream consumption, exchange, or degradation flux. Reversible enzymatic or transport reactions, such as glucose transport, glucose phosphorylation, and pyruvate–lactate conversion, were described using Michaelis–Menten rate laws. Lumped glycolytic conversion steps were represented by effective flux terms modulated by substrate saturation and enzyme-regulatory factors.

All the initial values of variables were set to the steady-state values under the reference condition unless otherwise indicated. The implications of variables and the initial values were provided in Supplementary Table S1. The standard settings of parameters are listed in Supplementary Table S2. Notably, we disrupted the coupling between EMT and glycolysis by modulating specific parameters in certain cases. The regulation of EMT by glycolysis was blocked by setting $k_{sz}=0$, $k_{z2}=0$, and enlarging $J_{HLAC}$ to a very large value (such as $10^{6}$), thereby removing glycolysis-associated activation of SNAIL/ZEB and lactate-dependent stabilization of HIF-1α. Conversely, the regulation of glycolysis by EMT was removed by setting $k_{0F}=0.7$, $k_{F}=0$, $k_{HK1}=0$, $k_{L2}=0$, and $k_{GLUTN}=0$, thereby cutting off SNAIL-, TWIST-, and N-Cad-associated regulation of FBP1, LDHA, HK, and GLUT1. In addition, parameter sensitivity was assessed through the relative variation in the threshold of $L_{O_{2}}$ corresponding to each bifurcation point when each parameter was increased or decreased by 10% (Supplementary Figures S1–S9). According to the results, our model is robust to fluctuations in most parameters while remaining sensitive to fluctuations in certain key parameters.

The system of ordinary differential equations (ODEs) was solved numerically using MATLAB R2026a (MathWorks, Natick, MA, USA) with the ode15s solver for stiff systems, which utilizes adaptive step-size control [59]. Bifurcation analysis was performed using Oscill8 v2.0.11 (https://oscill8.sourceforge.net/, accessed on 5 August 2026) to identify steady states, assess their stability, and map transitions between different phenotypic states as functions of $L_{O_{2}}$ and extracellular glucose availability. Phase portraits and time-series simulations were generated to visualize dynamic responses, hysteresis, and memory effects under various initial conditions and parameter sets.

## 3. Results

### 3.1. Dynamic Overview and Phenotypic Transitions in the Coupled EMT–Glycolysis Network

**Figure 2:**
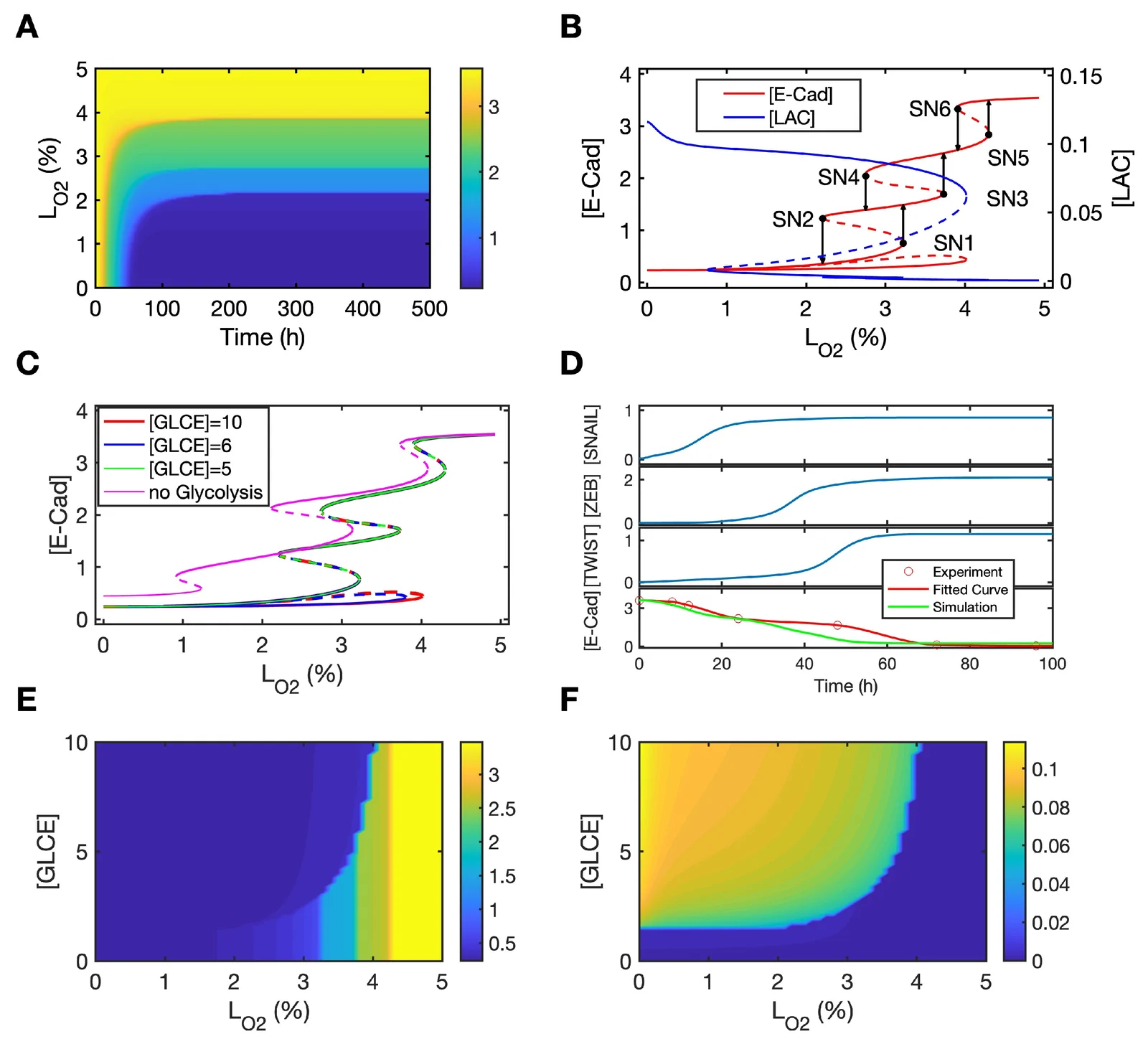
An overview of the EMT–glycolysis interplay under different conditions. (**A**) Phase diagram of [E-cadherin] as a function of time and $L_{O_{2}}\left(\%\right)$. (**B**) Bifurcation diagrams of [E-Cad] and [LAC] versus $L_{O_{2}}\left(\%\right)$. (**C**) Bifurcation diagrams of [E-Cad] at different [GLCE]. (**D**) Comparison of simulated temporal dynamics of EMT-TFs for $L_{O_{2}}\left(\%\right)=0.1\%$ with experimental data from MCF-7 cells [62]. Blue curves indicate simulated EMT-TF dynamics, the green curve indicates the simulated E-cadherin trajectory, red circles indicate experimental data, and the red curve indicates the fitted curve. For model comparison, all simulated trajectories were retained unchanged, while the experimental data were rescaled by aligning the initial experimental E-cadherin level with its corresponding initial value in simulations. (**E**,**F**) Phase diagrams showing the final steady state of [E-Cad] (**E**) and [LAC] (**F**) as a function of $L_{O_{2}}\left(\%\right)$ and [GLCE], highlighting a low-$L_{O_{2}}$/high-[GLCE] mesenchymal–glycolytic region. Of note, in the bifurcation diagrams, solid and dashed curves denote stable and unstable steady states, respectively. The default values of $L_{O_{2}}\left(\%\right)$ and [GLCE] were set to 0.1% and 10, respectively.

We first provide an overview of the dynamics and phenotypic transitions of the EMT–glycolysis system at various oxygen and glucose levels. The time courses of [E-Cad] for varying $L_{O_{2}}\left(\%\right)$ show that cells maintain a stable epithelial phenotype in normoxic or high-oxygen regions, whereas E-Cad decreases after $L_{O_{2}}$ enters the hypoxic range (Figure 2A) [23,60]. The response is threshold-like rather than linear: more severe hypoxia accelerates the transition from the epithelial to the mesenchymal state, but [E-Cad] eventually approaches a low-level plateau once the hypoxic stimulus is sufficiently severe [60,61].

Further, we reveal phenotypic transitions corresponding to EMT and metabolic reprogramming. The bifurcation diagram of [E-Cad] and [LAC] versus $L_{O_{2}}$ shows that decreasing $L_{O_{2}}$ drives a transition from the epithelial (E) to the mesenchymal (M) state through several bistable switches, while the steady-state lactate levels vary in a simple bistable switch with decreasing oxygen levels. This generates a distinct mesenchymal–glycolytic state by coupling low [E-Cad] to high lactate production, consistent with extensive evidence that EMT programs and metabolic rewiring are tightly interdependent in cancer [3,10].

The emergence and stability of this glycolytic state are critically dependent on glucose availability. As illustrated by the bifurcation diagrams under varying glucose levels (Figure 2C), extracellular glucose acts as a dose-dependent modulator of EMT dynamics. At lower concentrations (e.g., [GLCE] = 5), glucose mainly shifts the EMT induction threshold and sensitizes the system to hypoxic induction by lowering the $L_{O_{2}}$ threshold required to initiate EMT. Once glucose exceeds a critical range (e.g., transitioning from [GLCE] = 5 to [GLCE] = 6), the system undergoes a qualitative reorganization of its stability landscape. At sufficient concentrations ([GLCE] = 6 or 10), glucose not only promotes EMT but also creates an additional, stable mesenchymal–glycolytic branch. When the regulation of EMT by glycolysis is removed, the thresholds for EMT shift leftward, i.e., more severe hypoxia is required for EMT. Together, the promotion of EMT by glycolysis expands the stable parameter range of mesenchymal phenotypes and enhances hysteresis through high glycolytic flux and lactate accumulation [3,10,13].

To assess the biological validity of these predictions, we qualitatively compared the results from the core EMT module with experimental data [62]. The sequential induction of SNAIL, ZEB, and TWIST in response to hypoxia is consistent with the results of these EMT-TFs regulated under hypoxia across multiple cancer contexts (Figure 2D) [23,60,63,64]. Moreover, the dynamic curve of [E-Cad] at 0.1$\%\;O_{2}$ shows good agreement with experimental data [62]. Figure 2D provides a time-course comparison between simulated E-cadherin dynamics and experimentally reported E-cadherin changes under hypoxia. After normalization, the simulated trajectory shows overall agreement with the experimental data in both the decreasing trend and the approximate time scale of E-cadherin dynamics. This comparison supports the consistency between the predicted EMT dynamics and the reported experimental time course, although it should not be interpreted as a strict kinetic fit because the model parameters were not specifically fitted to this dataset.

The synergistic interplay between $L_{O_{2}}$ and glucose culminates in a well-defined cellular state landscape. The phase diagrams for final [E-Cad] (Figure 2E) and [LAC] levels (Figure 2F) reveal that $L_{O_{2}}$ and glucose cooperate to channel cells into a low-$L_{O_{2}}$/high-[GLCE] mesenchymal–glycolytic region. This region is defined by the dual signature of low [E-Cad] and high [LAC] production, corresponding to a metabolically active, invasion-associated phenotypic–metabolic state. This systems-level view demonstrates that oxygen and glucose jointly enhance the stability of EMT and glycolysis, in line with experimental studies [3,4].

### 3.2. Metabolic Feedback Reconstructs the Multi-Step Dynamics and Steady-State Structure of EMT

The progression of EMT is considered a multi-step process governed by a cascade of molecular events. We first dissected the canonical EMT dynamics in the absence of EMT regulation by glycolysis to establish a baseline and then demonstrated how glycolytic coupling reshapes this process, altering both the apparent transition order within this parameterization and the stability landscape.

**Figure 3:**
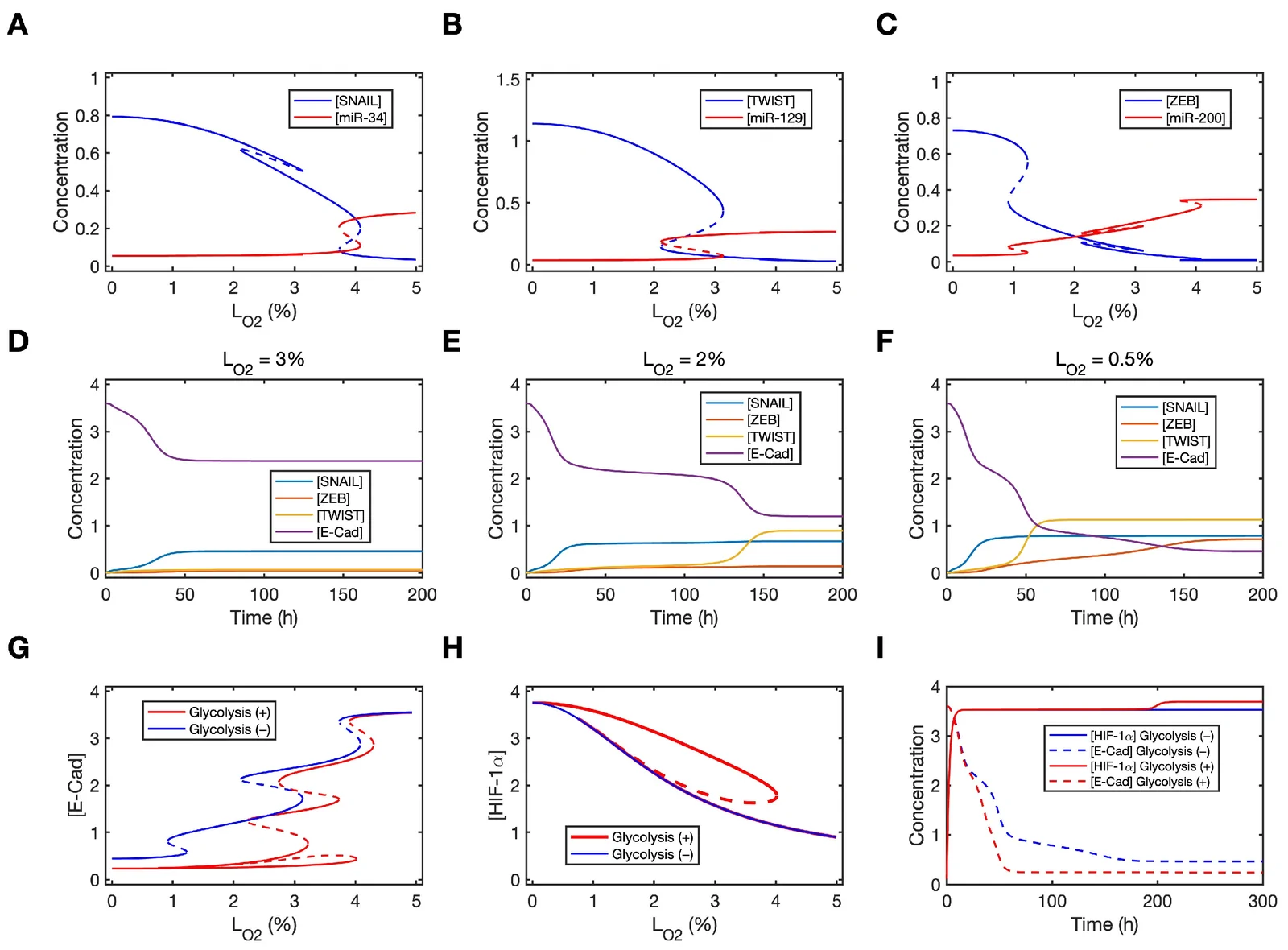
Glycolytic coupling rewires the multi-step dynamics of the canonical EMT program. (**A**–**C**) Bifurcation diagrams of the core EMT components versus $L_{O_{2}}$: SNAIL/miR-34 (**A**), TWIST/miR-129 (**B**), and ZEB/miR-200 (**C**), without the glycolytic feedback. (**D**–**F**) Dynamics of EMT components at different $L_{O_{2}}$, in the absence of the glycolytic feedback. (**G**) Bifurcation diagrams of [E-Cad] versus $L_{O_{2}}$. (**H**) Bifurcation diagrams of [HIF-1α] versus $L_{O_{2}}$. (**I**) Dynamics of [HIF-1α] under severe hypoxia and reoxygenation conditions. In panels (**G**–**I**), two cases of glycolytic feedback to EMT were considered, i.e., the absence or presence of the feedback. In the bifurcation diagrams, solid and dashed curves denote stable and unstable steady states, respectively.

In its core configuration, the EMT network operates as a series of multistable switches with decreasing $L_{O_{2}}$ regulated by the SNAIL/miR-34, TWIST/miR-129, and ZEB/miR-200 feedback loops (Figure 3A–C). For the EMT-TFs, SNAIL and TWIST rise to high levels near $2\;\%O_{2}$, while ZEB switches from moderate levels to high levels when $L_{O_{2}}$ further drops below $1\;\%O_{2}$. Due to the antagonism between each EMT-TF and the corresponding miRNA, they vary with decreasing $L_{O_{2}}$ in opposite manners. Correspondingly, cells undergo progressive EMT, switching from an epithelial (E) to the first hybrid E/M state, then to the second hybrid E/M state, and finally to a fully mesenchymal (M) state. Our results on the sequential activation of EMT-TFs are consistent with previous studies [16,25,32,65].

The temporal dynamics of the EMT-TFs’ concentrations and [E-cadherin] at distinct $L_{O_{2}}$ levels clearly illustrate this stepwise progression of EMT (Figure 3D–F). The sequential activation of SNAIL, TWIST, and ZEB leads to the gradual downregulation of [E-Cad], which drops to its lowest level only when all three EMT-TFs are activated. This stepwise activation program provides a canonical modeling framework for the multi-step nature of EMT [15,16]. However, this canonical sequence of EMT is profoundly reconfigured by metabolic coupling; the activation order of EMT-TFs could be remodeled (as shown in Figure 2D). Thus, the order of EMT-TF activation is context-dependent and can be reshaped by metabolic feedback and other factors [3,66].

The architectural impact of glycolysis on EMT progression is further elucidated by comparing the bifurcation diagrams with and without glycolytic feedback (Figure 3G). Only the thresholds for the glycolytically linked SNAIL/miR-34 and ZEB/miR-200 switches are significantly shifted rightward toward higher $L_{O_{2}}$ levels. The above results are consistent with the modular EMT circuit architecture established in previous theoretical and experimental studies [16,67].

Furthermore, glycolysis can feed back to HIF-1α to modulate EMT [13]. In the absence of glycolytic regulation, [HIF-1α] levels respond continuously to decreasing $L_{O_{2}}$. However, with glycolytic coupling, the HIF-1α system itself becomes bistable (Figure 3H). Once $L_{O_{2}}$ drops below a critical threshold (∼1%), high [lactate] levels from the now-active glycolytic state trigger a switch in HIF-1α to a high, self-sustaining state. The bifurcation diagrams indicate that the glycolytically coupled system requires a substantially higher $L_{O_{2}}$ to return to the low-HIF-1α/high-E-cadherin state during reoxygenation. Thus, glycolysis expands the hysteresis window and gives the system a memory of prior hypoxia (Figure 3I), maintaining the mesenchymal–glycolytic state across subsequent environmental shifts.

### 3.3. Glycolysis Modulates EMT Dynamics and Potentiates the Mesenchymal State

**Figure 4:**
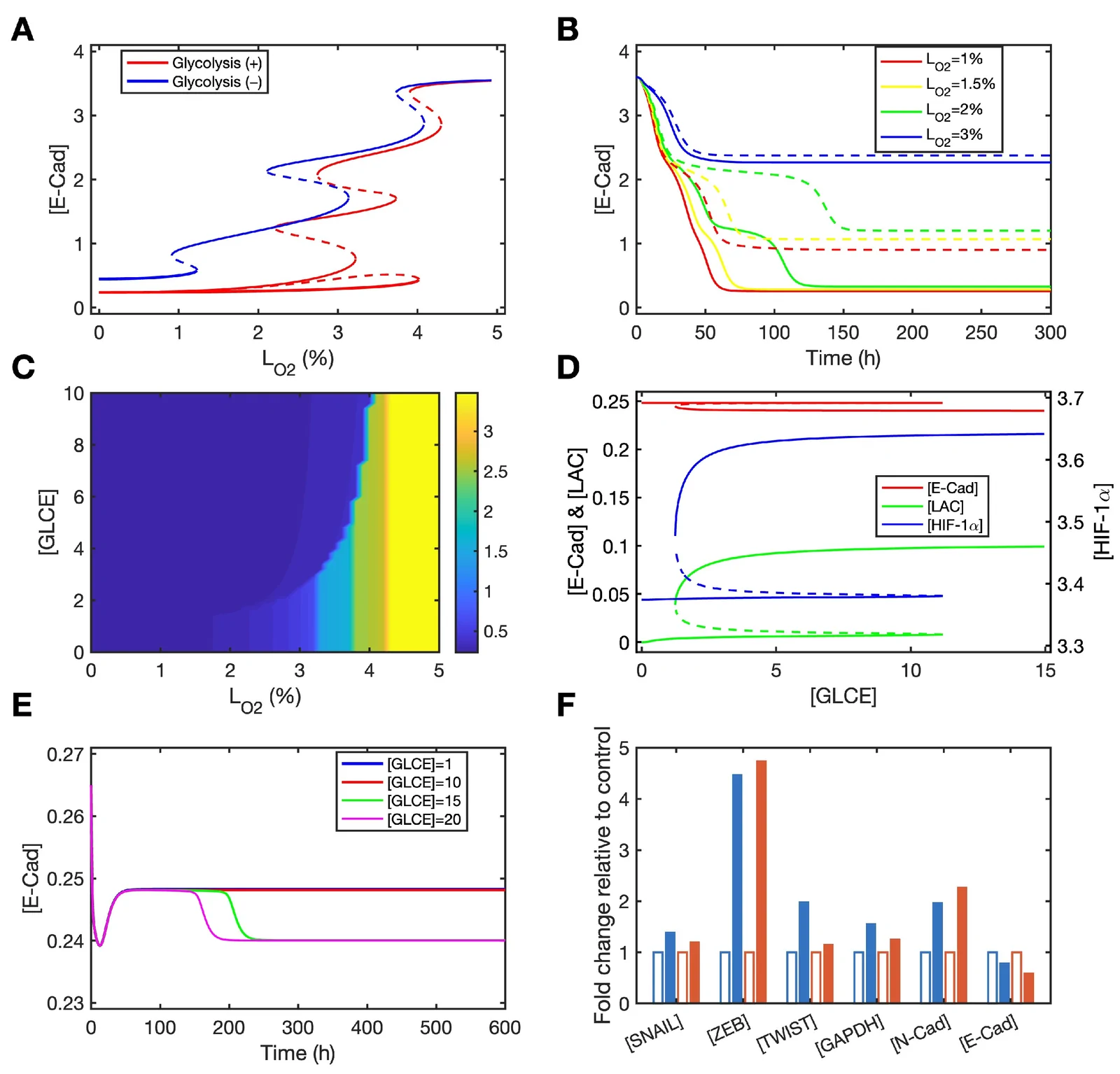
Glycolysis potentiates the EMT program by sensitizing and stabilizing the mesenchymal state. (**A**) Comparison of bifurcation diagrams of [E-Cad] versus $L_{O_{2}}$ with (red) and without (blue) glycolysis. (**B**) Dynamics of [E-Cad] in the presence or absence of EMT regulation by glycolysis at various $L_{O_{2}}$. (**C**) Phase diagram of the steady-state [E-cadherin] as a function of $L_{O_{2}}$ and [GLCE], with filled colors indicating the steady-state [E-cadherin] level. (**D**) Bifurcation diagrams of [E-cad], [LAC] and [HIF-1α] versus [GLCE]. (**E**) Temporal dynamics of [E-Cad] with different [GLCE]. (**F**) Model validation against experimental data from SCC9 cells by comparing fold changes in SNAIL, ZEB, TWIST, GAPDH, N-Cad, and E-Cad concentrations between non-glycolytic and glycolytic conditions [68]. $L_{O_{2}}\%$ values were set to 3.2% and 1% in the non-glycolytic and glycolytic cases, respectively. In panel (**F**), outlined bars indicate the non-glycolytic/control condition, and filled bars indicate the glycolytic condition. Of note, in the bifurcation diagrams, solid and dashed curves denote stable and unstable steady states, respectively.

We next investigated how glycolysis modulates the transition dynamics of EMT. We first compared the EMT bifurcation diagrams with and without EMT regulation by glycolysis (Figure 4A). With glycolytic coupling, the overall stability landscape shifts rightward to higher $L_{O_{2}}$. This indicates that glycolytically active cells can initiate and complete EMT under milder hypoxic conditions. Furthermore, glycolytic coupling significantly expands the hysteresis region of $L_{O_{2}}$ in the bifurcation diagram of [E-cad], rendering the mesenchymal state more resistant to reversal upon reoxygenation.

The effects of glycolysis on the temporal dynamics of EMT are further investigated. Time-series analysis under various $L_{O_{2}}$ levels demonstrates that for a given hypoxic stimulus (e.g., $L_{O_{2}}=1\%,1.5\%,2\%$), cells with active glycolysis exhibit faster and more complete downregulation of [E-cadherin] (Figure 4B). In contrast, cells lacking glycolytic feedback show a delayed or incomplete transition, often stabilizing in a hybrid E/M state. These results indicate that glycolysis serves not merely as a potentiator of EMT but also as a kinetic accelerator.

To capture the combined effects of $L_{O_{2}}$ and glucose, we mapped the steady-state level of [E-Cad] as a function of both parameters, starting from an epithelial state (Figure 4C). The color-coded diagram reveals an extensive region of the parameter space—particularly under low $L_{O_{2}}$ and high glucose—where cells stably reach a low-[E-Cad] basin. This broad stability basin for the mesenchymal phenotype underscores the synergistic coupling between hypoxia and glycolysis in establishing an invasion-associated mesenchymal–glycolytic state.

To further decouple these environmental factors, we analyzed the dose-dependent effect of glucose at a fixed oxygen level ($L_{O_{2}}=0.8\%$). The bifurcation diagram with glucose as the control parameter confirms that increasing glucose availability at a fixed $L_{O_{2}}$ can promote a switch-like activation of the high-glycolytic state (marked by a surge in [LAC]) and a concomitant downregulation of [E-Cad], thereby reinforcing the modeled stability structure (Figure 4D). Although the change in [E-Cad] level is modest, the stabilization effect is substantial, as evidenced by the wide bistable region of [HIF-1α]. Corresponding time-series simulations illustrate this clearly: cells exposed to higher levels of glucose transition to a more stable mesenchymal state, while glucose-limited cells remain in an epithelial or hybrid state (Figure 4E).

Finally, we compared the changes in the expression levels of the key markers of EMT and metabolism under glycolytic conditions with the corresponding experimental data [68]. The model qualitatively reproduces the directional changes observed experimentally: EMT-TFs (SNAIL, ZEB, and TWIST), the mesenchymal marker N-cadherin, and the glycolytic enzyme GAPDH increase, whereas E-cadherin decreases (Figure 4F). This directional agreement between simulations and experimental data supports the biological plausibility of our core hypothesis that the interplay between EMT and glycolysis constitutes a self-reinforcing circuit in cancer cells.

### 3.4. Glycolytic Coupling Modulates Hysteresis and EMT Reversibility

**Figure 5:**
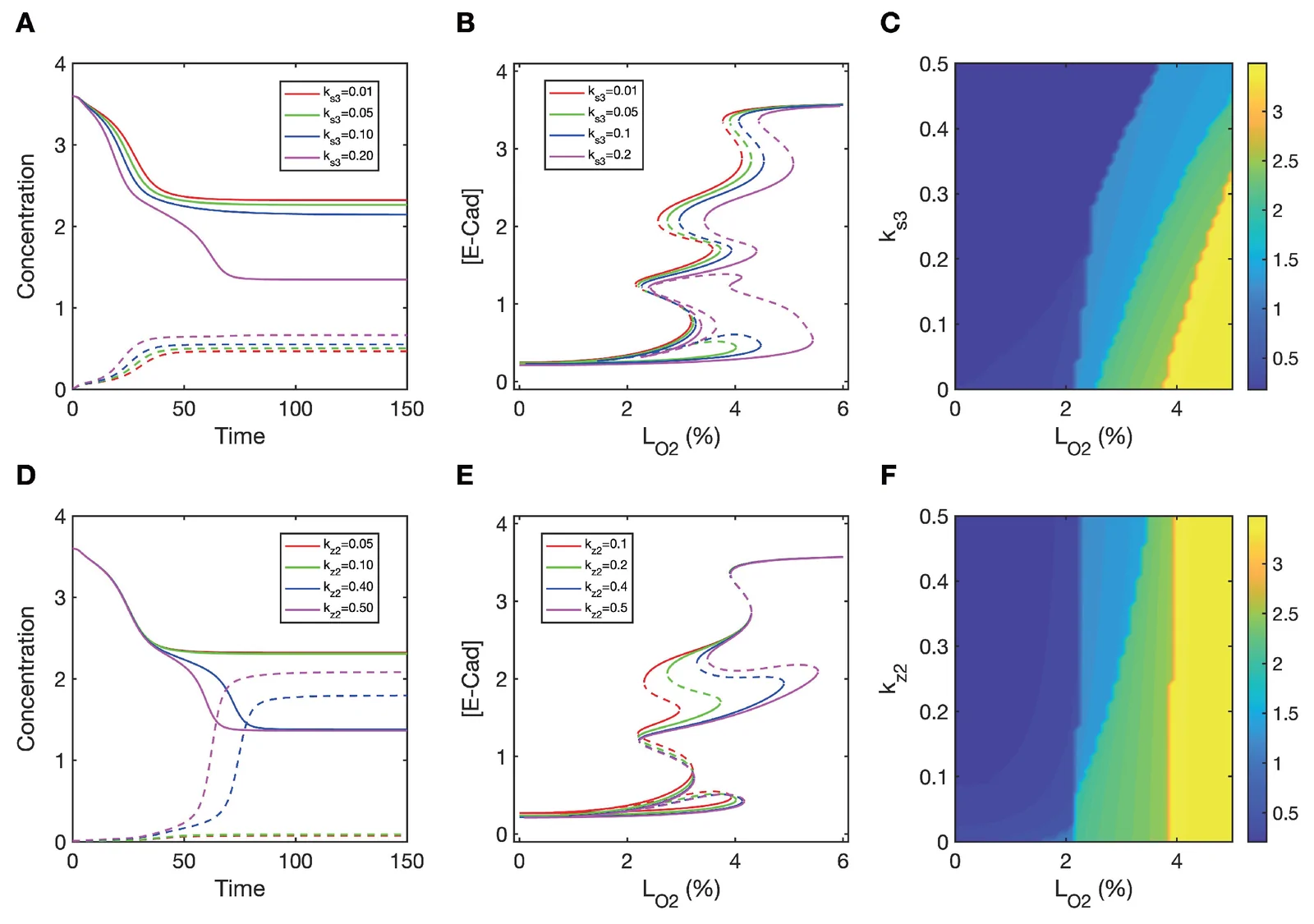
EMT regulation by glycolysis modulates its progression. (**A**–**C**) Effects of GAPDH-mediated SNAIL expression controlled by the promotion rate $k_{s3}$. (**A**) Time courses of [E-Cad] (solid lines) and [SNAIL] (dashed lines) at different $k_{s3}$. (**B**) Bifurcation diagrams of [E-Cad] versus $L_{O_{2}}$ at different $k_{s3}$. (**C**) The steady-state [E-Cad] as a function of $L_{O_{2}}$ and $k_{s3}$. (**D**–**F**) Effects of the LDHA–ZEB regulatory link controlled by the promotion rate $k_{z2}$. (**D**) Time courses of [E-Cad] (solid lines) and [ZEB] (dashed lines) at different $k_{z2}$. (**E**) Bifurcation diagrams of [E-Cad] versus $L_{O_{2}}$ at different $k_{z2}$. (**F**) The steady-state [E-Cad] as a function of $L_{O_{2}}$ and $k_{z2}$. In the bifurcation diagrams, solid and dashed curves denote stable and unstable steady states, respectively.

We investigated how specific metabolic–transcriptional feedback loops modulate the hysteresis and reversibility of the EMT program. We first focused on the GAPDH–SNAIL axis, a critical link that initiates the EMT cascade. By varying the promotion rate of SNAIL by GAPDH ($k_{s3}$), we observed a potent, dose-dependent acceleration of EMT kinetics. Higher $k_{s3}$ values lead to a faster and more complete downregulation of [E-Cad] even under mild hypoxia (Figure 5A). This kinetic advantage translates into a major architectural change in the stability landscape. The bifurcation diagram of [E-Cad] versus $L_{O_{2}}$, for different $k_{s3}$, reveals that increasing $k_{s3}$ systematically shifts the entire EMT transition structure towards higher $L_{O_{2}}$ levels, facilitating EMT under milder hypoxia (Figure 5B). Crucially, it also raises the $L_{O_{2}}$ threshold required for the mesenchymal-to-epithelial transition (MET), thereby expanding the hysteretic window in hypoxia-induced EMT. Moreover, the heatmap of [E-Cad] as a function of $k_{s3}$ and $L_{O_{2}}$ demonstrates that the GAPDH–SNAIL loop acts as a global stabilizer for the mesenchymal state, leveraging SNAIL’s upstream position in the EMT-TF hierarchy to fortify the entire network (Figure 5C).

Next, we examined the contribution of the LDHA–ZEB feedback loop, which governs a later stage of the EMT program. Similar to the GAPDH–SNAIL axis, increasing the promotion rate of ZEB by LDHA ($k_{z2}$) also accelerates EMT via the decrease in [E-Cad] and facilitates a deeper mesenchymal transition (Figure 5D). However, its impact on the system’s hysteresis is more nuanced. Bifurcation analysis shows that increasing $k_{z2}$ preferentially stabilizes a later or intermediate mesenchymal-like branch in EMT, making reversal more difficult even when $L_{O_{2}}$ rises substantially (Figure 5E). Unlike SNAIL, ZEB has limited feedback influence on other EMT-TFs in our network. Consequently, the LDHA–ZEB loop functions as a more localized stabilizer, primarily reinforcing the ZEB/miR-200 switch without globally propagating its stabilizing effect throughout the entire network, a finding consistent with the parameter-space landscape (Figure 5F).

Taken together, these results dissect the specific contributions of individual metabolic–transcriptional feedback loops to the overall system’s stability. Among these routes, the lactate–HIF-1α loop provides the most direct system-level coupling between glycolytic activity and hypoxia-responsive EMT regulation, thereby expanding the hysteresis window and supporting phenotypic memory. By contrast, the GAPDH-SNAIL and LDHA-ZEB regulatory links mainly act within the EMT-TF regulatory layer. The GAPDH–SNAIL axis acts as a master initiator and global stabilizer, while the LDHA–ZEB axis serves as a stage-specific reinforcer. This hierarchical and modular stabilization mechanism, driven by specific glycolytic enzymes, provides a quantitative explanation for the strong hysteresis and memory observed in the coupled EMT–glycolysis system.

To quantitatively validate this targeted feedback, a global parameter sensitivity analysis on steady-state E-cadherin expression was performed (Supplementary Figure S1). The results reveal a distinct hierarchy: while core EMT-TF parameters dominate and general glycolytic reaction rates exert negligible retrograde influence, specific coupling nodes—such as the LDHA-mediated ZEB promotion rate ($k_{z2}$) and LDHA degradation rate ($k_{dL}$)—significantly alter the epithelial status. This mathematically corroborates that metabolic feedback to EMT is driven by highly targeted control nodes rather than generic energy flux.

### 3.5. Bistable Switch in Glycolysis

The profound impact of glycolysis on EMT dynamics suggests that the glycolytic network itself possesses complex, non-linear properties. We therefore dissected this metabolic subsystem to reveal its intrinsic operational logic. Our analysis demonstrates that the core glycolytic pathway, driven by a key positive feedback architecture, can function as a robust bistable switch [69]. This switch is capable of locking cells into either a low- or high-glycolytic state depending on substrate availability and cellular history.

**Figure 6:**
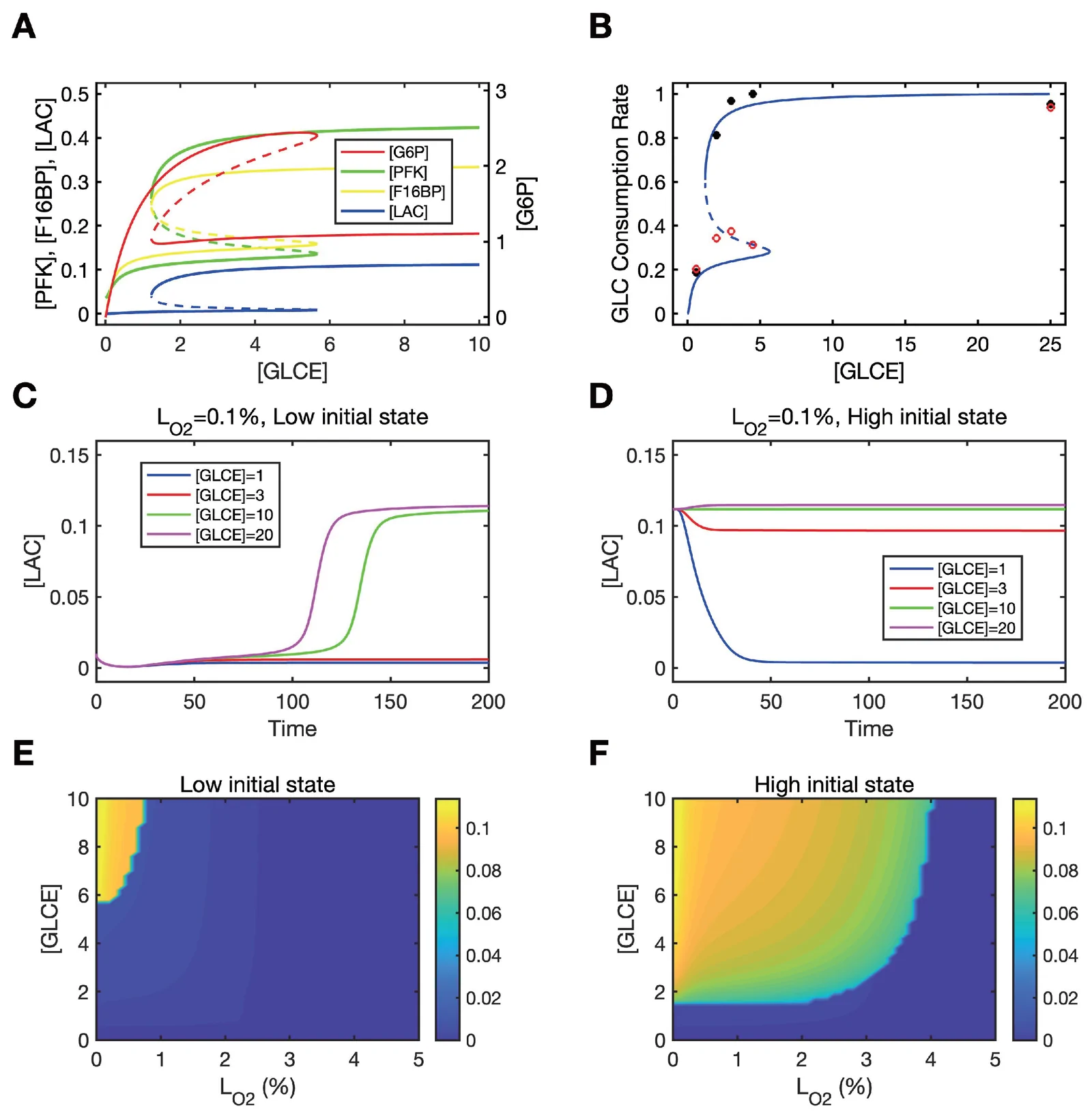
The glycolytic subsystem functions as a bistable switch. (**A**) Bifurcation diagram of key glycolytic metabolites versus [GLCE]. (**B**) Comparison of the predicted bistable glucose consumption rate with experimental data [69]. Circles indicate experimental data points, and the fitted/predicted curve indicates the model comparison. (**C**,**D**) Temporal dynamics of [LAC] from low (**C**) and high (**D**) initial states. (**E**,**F**) Phase diagrams of the final [Lactate] state starting from low (**E**) and high (**F**) initial conditions. In the bifurcation diagrams, solid and dashed curves denote stable and unstable steady states, respectively.

The bistability of the glycolytic switch is rooted in the upstream PFK-centered allosteric regulation and the downstream pyruvate–lactate branch. In particular, modeling and kinetic analysis have indicated that the feedback loop involving fructose-1,6-bisphosphate and glycolytic control nodes can generate multiple steady states in glycolytic flux [69,70]. Bifurcation analysis with extracellular glucose GLCE as the control parameter reveals that this feedback architecture generates a classic bistable response (Figure 6A). As [GLCE] increases, the system remains in a low-glycolytic state until it surpasses a critical threshold, at which point key metabolites and the final product [LAC] abruptly switch to a high-concentration steady state. This switch-like behavior of glycolytic flux is consistent with experimentally grounded systems interpretations of glycolytic control in proliferative contexts [69], which similarly exhibit a distinct bistable characteristic (Figure 6B).

This bistability endows the system with a profound dependency on its initial conditions, a hallmark of hysteretic switches. We illustrated this by simulating the temporal evolution of [lactate] from either a low- or high-glycolytic initial state under fixed hypoxia ($L_{O_{2}}=0.1\%$) (Figure 6C,D). At an intermediate glucose concentration (e.g., [GLCE] = 3), the final state of the system is entirely determined by its initial state: cells beginning in a low-glycolytic state remain low, while those starting high remain high. Thus, at the same [GLCE], the system remembers its initial metabolic conditions rather than responding only to the current glucose concentration. This demonstrates the existence of two distinct basins of attraction, separated by an unstable steady state.

To generalize this behavior, we mapped the final steady state of [LAC] across the full parameter space of $L_{O_{2}}$ and glucose, starting from both low and high initial conditions (Figure 6E,F). The resulting phase diagrams clearly delineate a large region of bistability where the final metabolic state is history-dependent. Comparing the two diagrams reveals the vast hysteretic window created by the intrinsic dynamics of the glycolytic switch. This analysis confirms that the glycolytic subsystem does not merely respond passively to substrate levels but operates as an autonomous, history-dependent switch. This intrinsic bistable nature is the fundamental reason why glycolysis can so powerfully modulate the stability and dynamics of the coupled EMT program, as observed in the previous sections.

### 3.6. EMT Actively Drives Glycolysis and the Warburg Effect

Having established the bidirectional coupling between EMT and glycolysis, we next dissected the reverse arm of this circuit: how the EMT program actively reprograms cellular metabolism. Our model indicates that EMT mainly promotes glycolysis by upregulating lactate production, consistent with the general principle that EMT-TFs can rewire metabolic priorities to support migration, invasion, and survival under stress [3,10]. In the following, we investigate the effects of the EMT pathway on glycolysis induction.

**Figure 7:**
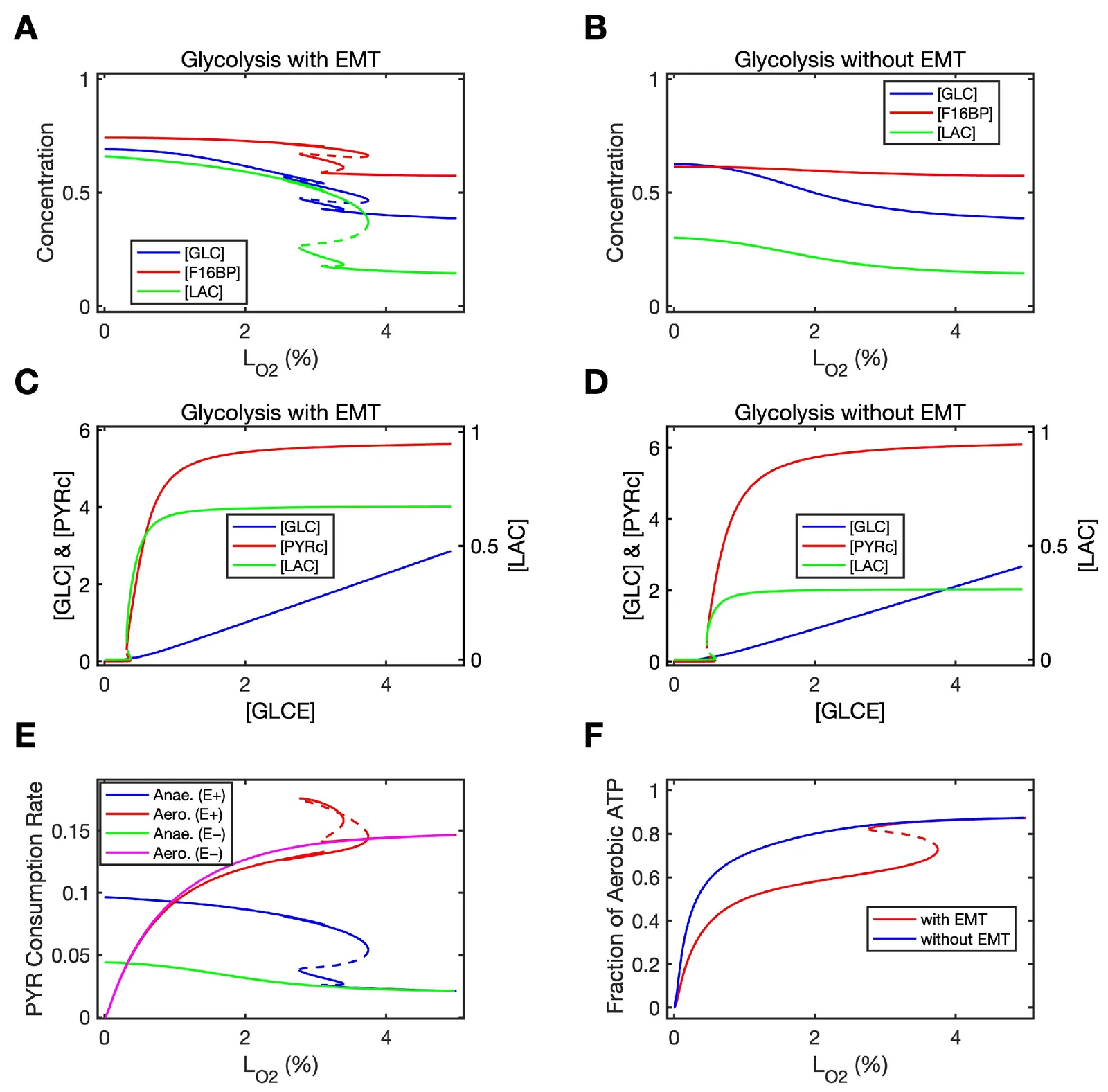
EMTactively promotes metabolic reprogramming toward a Warburg-like, lactate-producing state. (**A**,**B**) Bifurcation diagrams of steady-state levels of glycolytic components versus $L_{O_{2}}$ with (**A**) and without (**B**) EMT feedback. (**C**,**D**) Response curves of the downstream components of pyruvate to [GLCE] with (**C**) or without (**D**) EMT feedback. (**E**) Analysis of pyruvate consumption flux in the presence (E+) or absence (E−) of EMT feedback. (**F**) The proportion of ATP generated via the simplified aerobic route in cells experiencing EMT. In the bifurcation diagrams, solid and dashed curves denote stable and unstable steady states, respectively.

The glycolytic metabolite profiles with and without EMT feedback are distinct. In cells that have undergone EMT, the concentrations of upstream glycolytic intermediates, such as intracellular glucose ([GLC]) and fructose-1,6-bisphosphate ([F16BP]), show only modest changes in response to hypoxia or elevated glucose (Figure 7A,C). This contrasts with the much stronger accumulation of the final product, [LAC]. The bifurcation diagrams for cells lacking EMT regulation show a similar, weak response for all metabolites, including lactate (Figure 7B,D). Thus, EMT does not uniformly amplify all glycolytic intermediates; its dominant effect is concentrated on the components associated with lactate production. Indeed, the concentration of pyruvate ([PYR_c_]), the immediate precursor to lactate, remains largely unchanged by EMT status (compare Figure 7C,D). Together, these results indicate that the primary metabolic effects of EMT mainly contribute to enhancing the pyruvate-to-lactate conversion.

This hypothesis is directly examined by analyzing the metabolic flux at the pyruvate branch point. We examined the rates of pyruvate consumption through either anaerobic glycolysis (conversion to lactate) or a simplified aerobic route (entry into the TCA cycle). In EMT-positive cells, the lactate-producing route is strongly elevated across $L_{O_{2}}$ levels, while the simplified aerobic route shows no corresponding enhancement (Figure 7E). This metabolic rewiring is consistent with a Warburg-like shift: a relative preference for lactate production even in the presence of oxygen. Consequently, the relative fraction of ATP generated via the modeled aerobic route is lower in EMT cells compared to their epithelial counterparts (Figure 7F) [71]. However, because our model does not include the detailed pathway for OXPHOS, the above results do not exclude hybrid glycolysis/OXPHOS phenotypes that have been reported in metastatic and therapy-resistant cancer cells [19,72].

Our results indicate that EMT actively drives metabolic reprogramming by modulating a key regulatory point in central carbon metabolism. By upregulating lactate dehydrogenase (LDHA), the EMT program introduces a strong lactate-producing pathway at the pyruvate branch point, thereby reducing the relative aerobic ATP fraction in our model. This supports the mesenchymal–glycolytic state and reinforces lactate-mediated HIF-1α feedback, thereby closing the self-sustaining loop between phenotype and metabolism. A comprehensive treatment of OXPHOS plasticity will require an expanded mitochondrial module.

### 3.7. A Self-Sustaining Feedback Loop Between EMT and Glycolysis Produces a Robust Bistable Switch

The bidirectional crosstalk between EMT and glycolysis produces a self-sustaining positive feedback loop. This feedback loop acts as a robust, system-level bistable switch, capable of maintaining a mesenchymal–glycolytic state after the initial inducing signal is removed. This mechanism provides a quantitative basis for phenotypic “memory” and persistent invasion-associated plasticity, both of which are increasingly linked to stable EMT and metabolic adaptation [3,4,66]. The existence of this system-level bistability is illustrated by comparing the final state landscapes starting from either an epithelial (E) or a mesenchymal (M) initial condition. This emergent dynamic behavior is consistent with the known capacity of core EMT miRNA–TF circuits to generate multistability and hysteresis at the phenotype level [16,67], as well as evidence that lactate-mediated reinforcement of HIF-1α provides an additional stabilizing arm for invasion-associated states [13].

**Figure 8:**
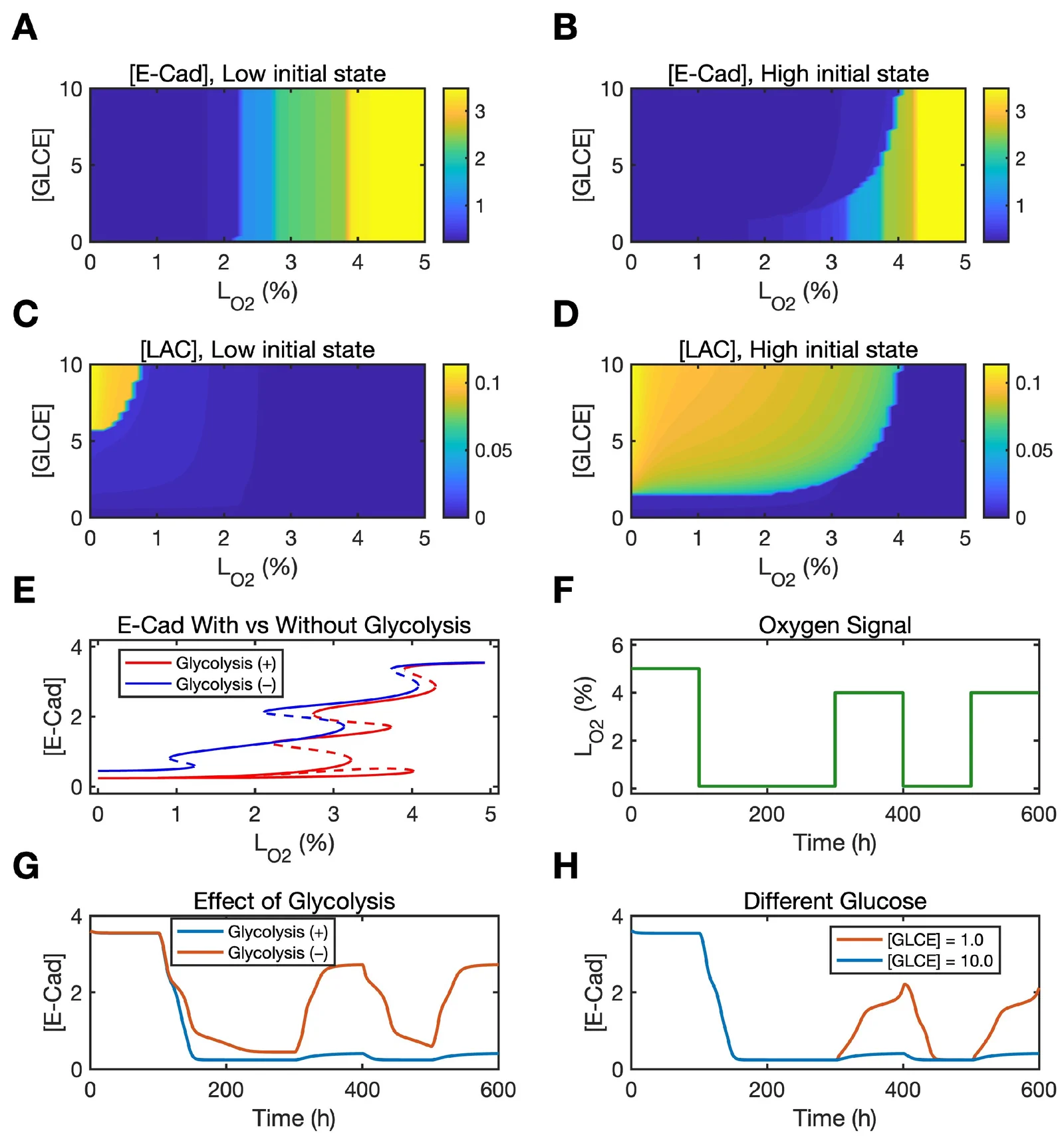
The self-sustaining EMT–glycolysis loop confers robust memory under dynamic oxygen signals. (**A**–**D**) Phase diagrams of final [E-Cad] (**A**,**B**) and [LAC] (**C**,**D**) from epithelial (**A**,**C**) and mesenchymal (**B**,**D**) initial states, illustrating a large region of system-level bistability. (**E**) Bifurcation diagram of [E-Cad] versus $L_{O_{2}}$ in the presence or absence of EMT regulation by glycolysis. (**F**) The applied oscillatory $L_{O_{2}}$ signal protocol. (**G**,**H**) Temporal response of [E-cadherin] to the oxygen signal.

Starting from different initial states, the phase diagrams for both [E-Cad] and [LAC] reveal a large region in the $L_{O_{2}}$–glucose parameter space where the system’s final state, marked by either [E-Cad] or [LAC], is history-dependent (Figure 8A–D). This hysteresis is the macroscopic signature of the underlying self-reinforcing loop. The bifurcation diagrams of [E-Cad] versus $L_{O_{2}}$ show that the glycolytic regulation shifts several thresholds of $L_{O_{2}}$ rightward (Figure 8E). Thus, the regulation of EMT by the glycolytic pathway potentiates the maintenance of the mesenchymal-like state at higher oxygen levels.

We mimicked the fluctuating hypoxic microenvironment as a temporal series of flat-wave oxygen signals. We subjected the cells to an oxygen cycling protocol: starting from a high-oxygen/normoxic-like condition ($L_{O_{2}}=5\%$), followed by severe hypoxia ($L_{O_{2}}=0\%$), then alternating between partial reoxygenation ($L_{O_{2}}=4\%$) and severe hypoxia in a cyclical pattern (Figure 8F). In the absence of glycolytic feedback, [E-Cad] levels follow the variations in $L_{O_{2}}$ and recover during each reoxygenation phase (Figure 8G). In the normal case with EMT regulation by glycolysis, the initial severe hypoxic episode is sufficient to push the cell into a low-[E-Cad] mesenchymal–glycolytic state. During subsequent reoxygenation phases, even when $L_{O_{2}}$ recovers to 4%, [E-cadherin] remains low. This demonstrates that the EMT–glycolysis loop can convert dynamic oxygen fluctuations into persistent mesenchymal memory under glucose-sufficient conditions.

This memory and state-locking effect is critically dependent on the metabolic arm of the feedback loop. When the same simulation is run with limited glucose ([GLCE] = 1.0), the self-sustaining circuit fails to engage. The cell is unable to maintain the mesenchymal state upon reoxygenation and recovers toward a higher-E-cadherin epithelial-like state (Figure 8H). This confirms that sufficient glycolytic flux is required for long-term mesenchymal memory under fluctuating oxygen conditions.

Taken together, the coupled EMT–glycolysis network functions as a robust, history-dependent switch. It allows transient hypoxic cues to be stored as persistent mesenchymal memory, provided that sufficient glucose is available to sustain the metabolic arm of the feedback loop. This provides a systems-level mechanism by which a fluctuating microenvironment can be transformed into durable mesenchymal–glycolytic states and long-lived invasion-associated phenotypic plasticity.

## 4. Discussion and Conclusions

The epithelial–mesenchymal transition (EMT) and aerobic glycolysis are two fundamental processes that connect the tumor microenvironment to invasion-associated phenotypic plasticity [1,2,4,10,73]. In the broader context of tumor evolution, tumor cells are continuously shaped by fluctuating microenvironmental factors rather than by static conditions. This study focuses on the interplay between hypoxia-driven EMT and glucose-dependent glycolysis. We developed an integrated model of the EMT–glycolysis network and exploited it to determine how the levels of oxygen and glucose modulate cell phenotypes. The model shows that hypoxia and glucose jointly define the regions in which epithelial, hybrid, and mesenchymal–glycolytic phenotypes are stable.

A key conclusion of this work is that glycolysis is not merely a passive downstream consequence of EMT. Although previous EMT models captured the multi-step and multistable nature of transcriptional circuits such as SNAIL/miR-34 and ZEB/miR-200, they often omit the metabolic dimension [16,32,34,65]. Our model indicates that activation of a high-glycolytic state introduces an additional stable mesenchymal–glycolytic branch, expands the oxygen–glucose parameter region supporting low-E-cadherin states, and enhances the stability of hybrid phenotypic–metabolic states [3,69]. In this sense, glycolysis acts as a global regulator of the EMT state space. Furthermore, we showed that this metabolic feedback selectively sensitizes specific EMT modules (such as SNAIL and ZEB) to hypoxia. It reshapes not only the threshold for EMT induction but also the persistence and reversibility of the resulting phenotypes [61].

**Figure 9:**
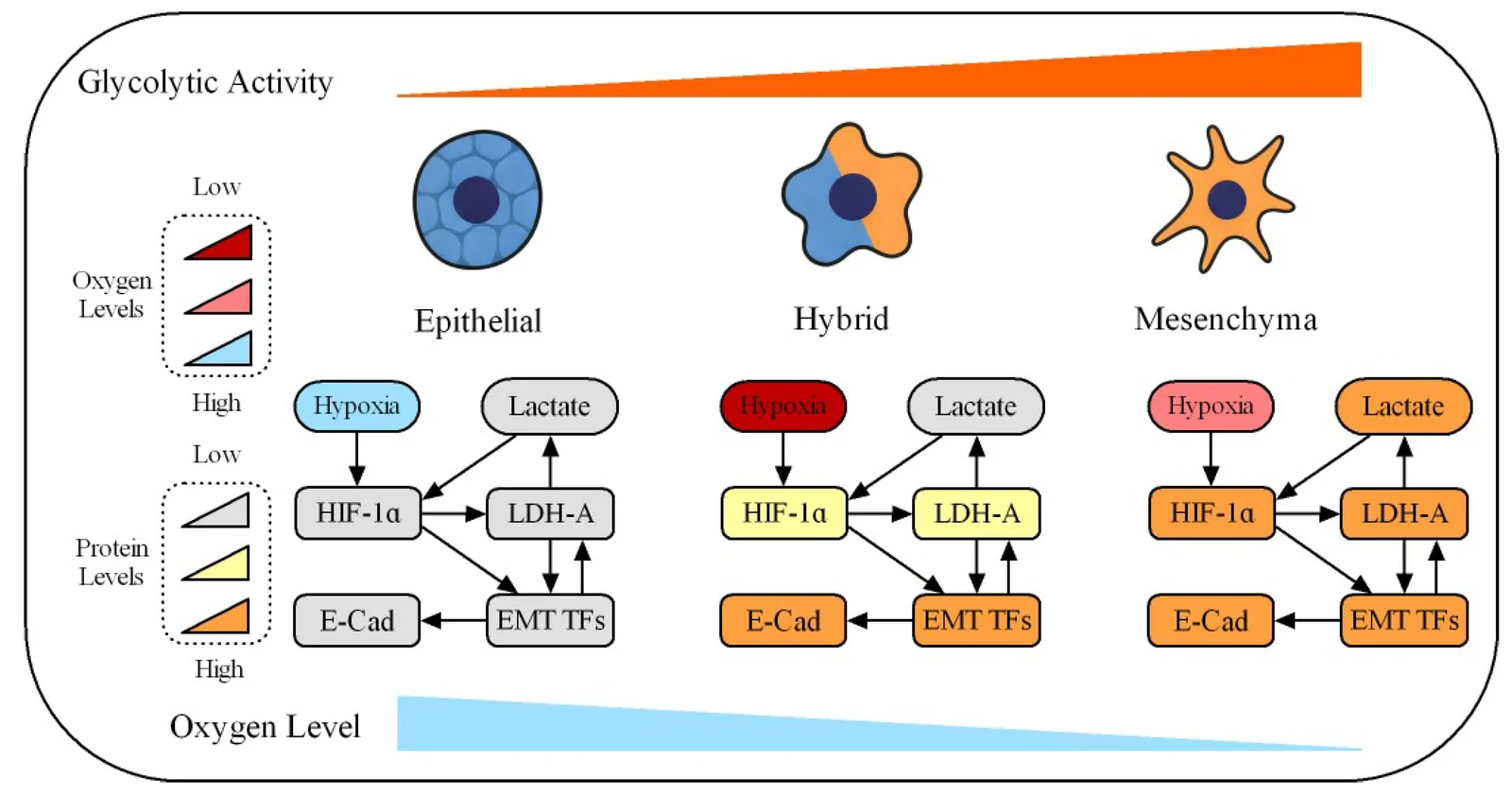
A schematic diagram of the self-sustaining EMT–glycolysis feedback loop and its role in phenotypic memory. The simplified network shows the core regulatory interactions: hypoxia activates HIF-1α, which promotes EMT-TFs and glycolysis; EMT-TFs drive EMT and enhance glycolysis; glycolysis produces lactate, which stabilizes HIF-1α, forming a positive feedback loop. Three physiological scenarios are illustrated: a normoxic epithelial state with weak EMT and basal glycolysis; a hypoxia-dependent state in which EMT and glycolysis remain partially activated only while the external hypoxic signal persists; and a feedback self-sustaining state in which sufficient glucose supports high glycolytic flux, lactate accumulation, and persistent EMT activation even after the initial hypoxic input is removed. This bistable switch mechanism explains how transient environmental stimuli can be converted into persistent, invasion-associated cellular phenotypes that resist simple reoxygenation.

The central dynamical mechanism identified here is a self-sustaining positive feedback loop between EMT and glycolysis (Figure 9). In one direction, EMT promotes a Warburg-like lactate-producing bias through enhanced LDHA activity; in the other direction, glycolysis stabilizes EMT through lactate–HIF-1α reinforcement and glycolytic-enzyme-mediated potentiation of EMT-TF activity [3,10,13,74]. The macroscopic consequence is hysteresis and phenotypic memory. Our simulations show that a transient hypoxic episode can be converted into a stable mesenchymal–glycolytic state when glucose is sufficient, whereas the same hypoxic input fails to produce durable locking when glucose is limited. This finding assigns a decisive role to metabolic context: glucose availability determines whether a transient environmental stress remains reversible or is stored as long-lived mesenchymal memory [3,16,73]. This behavior is also consistent with the Warburg effect, in which tumor cells can maintain elevated glycolytic activity even under oxygen-sufficient conditions [10,75]. This finding has significant implications for understanding the origins of constitutively mesenchymal and therapy-resistant cells within a tumor, which often arise from transient exposure to hypoxic or inflammatory niches [61,73,76]. Nevertheless, this prediction should not be interpreted as a universal property of all cancer cells and requires further experimental validation.

Our analysis therefore defines three dynamical regimes for the coupled EMT–glycolysis system. Under normoxia with basal metabolic demand, cells remain epithelial and weakly glycolytic. Under hypoxia, EMT and glycolysis are progressively activated depending on the strength of the signal; however, if the metabolic feedback arm is insufficient, this activation remains reversible and requires continuous environmental signaling for its maintenance [61,74]. Under hypoxia combined with adequate glucose, the system enters a feedback self-sustainment regime in which the components in the EMT pathway and glycolytic pathway mutually reinforce each other. Thus, a qualitatively distinct mesenchymal–glycolytic phenotype appears in which low E-cadherin and high lactate coexist, and the phenotype becomes resistant to simple reoxygenation. The transition between these regimes is governed by thresholds in both oxygen levels and glucose availability, consistent with the ability of EMT and metabolic circuits to generate multistability and hysteresis [3,16,69]. It should be emphasized that sufficient glucose is required to generate a stable mesenchymal–glycolytic state.

From a practical standpoint, these findings suggest that interventions aimed at reversing EMT transcriptional programs alone may be insufficient when the glycolytic feedback loop to EMT remains intact. The model predicts that disrupting both the EMT regulatory layer and the glycolytic support layer should more effectively destabilize the mesenchymal–glycolytic state. Candidate intervention points include EMT regulators such as SNAIL or ZEB and metabolic nodes such as LDHA, PFK, or the lactate–HIF-1α reinforcement axis. Because glucose availability acts as a threshold-like modulator, metabolic context may also determine when EMT-targeted interventions are most effective [2,10,66]. These predictions require strict experimental validation in the future. The robust bistability we predict suggests that cancer cell populations exposed to fluctuating microenvironmental conditions will develop persistent heterogeneity, with subpopulations locked into different stable states [2,73]. This heterogeneity is a major driver of therapy resistance, as different cell subsets may respond differently to treatments [73,76]. Our model provides a framework for understanding and potentially predicting this heterogeneity based on the metabolic and oxygenation history of the tumor [3,61].

Several limitations of the current study should be acknowledged. First, the model focuses on the transcriptional–metabolic axis and omits the earliest junctional events, such as hypoxia-induced miRNA regulation of tight junctions [77,78]. E-cadherin is used as a compact epithelial readout rather than a universal surrogate of EMT, because its downregulation is not observed in all hypoxic contexts [79]. Second, other pathways, such as Hsp70-mediated proteostasis [80] and TGF-β signaling, may also shift the predicted thresholds of oxygen and glucose levels. Moreover, the critical role of glucose availability in EMT is likely to be context-dependent. It has been reported that high glucose cannot induce EMT marked by the loss of E-cadherin in some cases [80,81]. These findings indicate that glucose availability alters the EMT–metabolic stability landscape, but the ultimate cellular phenotype depends on additional factors beyond those included in our model. In addition, we consider only the lactate–HIF-1α feedback branch in the simplified metabolic module; pathways involving OXPHOS, AMPK signaling, and ROS should be included to fully capture hybrid metabolic plasticity [2,3,72]. Taken together, a more comprehensive model is required to reconcile discrepancies regarding the interplay between EMT and glycolysis across different experimental studies.

In conclusion, this work clarifies how hypoxia and glucose availability jointly shape the EMT–glycolysis stability landscape. We show that glycolytic reinforcement can broaden mesenchymal stability, stabilize hybrid phenotypic–metabolic states, and transform transient hypoxia into persistent mesenchymal memory. The model may offer a systems-level explanation for how fluctuations in the metabolic microenvironment can drive durable invasion-associated phenotypic evolution. The coupled EMT–glycolysis axis therefore represents both a mechanistic basis for cancer cell plasticity and a theoretical foundation for strategies designed to prevent or reverse malignant phenotypes [3,16,69].

## Data Availability

The original contributions presented in this study are included in the article/Supplementary Material [15,17,20,69,82,83,84,85,86,87,88,89,90,91,92,93,94,95]. Further inquiries can be directed to the corresponding authors. Additionally, the ordinary differential equations (ODEs) used in this study are provided in Supplementary Material S1, along with example scripts for numerical integration that rely solely on standard built-in solvers in MATLAB R2026a (MathWorks, Natick, MA, USA) and Oscill8 v2.0.11. No custom algorithms were developed beyond the configuration of the equations.

## Supplementary Materials

The following supporting information can be downloaded at https://www.mdpi.com/article/10.3390/cimb48090907/s1: Section S1: Model equations; Section S2: Model variables and baseline settings; Table S1: State variables and initial conditions; Table S2: Baseline parameter settings for the coupled EMT–glycolysis model; Section S3: Parameter sensitivity analysis; Figures S1–S9: Parameter sensitivity analyses of steady-state E-cadherin expression and saddle-node bifurcation points in the EMT–glycolysis model.
