# Supplementary material for: Mechanisms Underlying the Coordination of EMT and Glycolysis Mediated by Hypoxia and Glucose
Source: Curr Issues Mol Biol. 2026 Sep 4;48(9):907. doi: 10.3390/cimb48090907 (PMC13604997; doi:10.3390/cimb48090907)
Supplement: Supplementary file 1 [file cimb-48-00907-s001.zip › cimb-4517036-supplementary.pdf]

Supplementary Materials

S1 Model equations

The coupled EMT–glycolysis model is formulated as the following system of ordinary differential equations. Square brackets denote the concentration or abundance of the corresponding molecular species.

$$\frac{d\,[\mathrm{HIF1}\alpha]}{dt} = k_{0\mathrm{H}} - \left(k_{d0\mathrm{H}} + k_{d\mathrm{H}} \frac{\left(\frac{[\mathrm{O_2}]}{J_{\mathrm{H}}}\right)^{n_{\mathrm{H}}}}{\left(1 + \left(\frac{[\mathrm{O_2}]}{J_{\mathrm{H}}}\right)^{n_{\mathrm{H}}}\right)\left(1 + \left(\frac{[\mathrm{LAC}]}{J_{\mathrm{HLAC}}}\right)^{n_{\mathrm{HLAC}}}\right)} \right) [\mathrm{HIF1}\alpha] \tag{S1}$$

$$\begin{aligned}\frac{d\,[\mathrm{snail}]}{dt} = k_{0\mathrm{s}} + k_{\mathrm{s}} \Bigg(& k_{\mathrm{s1}} \frac{\left(\frac{[\mathrm{TWIST}]}{J_{\mathrm{sT}}}\right)^{n_{\mathrm{sT}}}}{1 + \left(\frac{[\mathrm{TWIST}]}{J_{\mathrm{sT}}}\right)^{n_{\mathrm{sT}}}} + k_{\mathrm{s2}} \frac{\left(\frac{[\mathrm{HIF1}\alpha]}{J_{\mathrm{sH}}}\right)^{n_{\mathrm{sH}}}}{1 + \left(\frac{[\mathrm{HIF1}\alpha]}{J_{\mathrm{sH}}}\right)^{n_{\mathrm{sH}}}} \\ & + k_{\mathrm{s3}} \frac{\left(\frac{[\mathrm{GAPDH}]}{J_{\mathrm{sG}}}\right)^{n_{\mathrm{sG}}}}{1 + \left(\frac{[\mathrm{GAPDH}]}{J_{\mathrm{sG}}}\right)^{n_{\mathrm{sG}}}} \Bigg) - k_{d\mathrm{s}}[\mathrm{snail}]\end{aligned} \tag{S2}$$

$$\frac{d\,[\mathrm{SNAIL}]}{dt} = k_{\mathrm{S}} \frac{[\mathrm{snail}]}{1 + \left(\frac{[\mathrm{miR\text{-}34}]}{J_{\mathrm{SR3}}}\right)^{n_{\mathrm{SR3}}}} - k_{d\mathrm{S}}[\mathrm{SNAIL}] \tag{S3}$$

$$\frac{d\,[\mathrm{zeb}]}{dt} = k_{0\mathrm{z}} + k_{\mathrm{z}} \left(k_{\mathrm{z1}} \frac{\left(\frac{[\mathrm{SNAIL}]}{J_{\mathrm{zS}}}\right)^{n_{\mathrm{zS}}}}{1 + \left(\frac{[\mathrm{SNAIL}]}{J_{\mathrm{zS}}}\right)^{n_{\mathrm{zS}}}} + k_{\mathrm{z2}} \frac{\left(\frac{[\mathrm{LDHA}]}{J_{\mathrm{zL}}}\right)^{n_{\mathrm{zL}}}}{1 + \left(\frac{[\mathrm{LDHA}]}{J_{\mathrm{zL}}}\right)^{n_{\mathrm{zL}}}} \right) - k_{d\mathrm{z}}[\mathrm{zeb}] \tag{S4}$$

$$\frac{d\,[\mathrm{ZEB}]}{dt} = k_{\mathrm{Z}} \frac{[\mathrm{zeb}]}{1 + \left(\frac{[\mathrm{miR\text{-}200}]}{J_{\mathrm{ZR2}}}\right)^{n_{\mathrm{ZR2}}}} - k_{d\mathrm{Z}}[\mathrm{ZEB}] \tag{S5}$$

$$\frac{d\,[\mathrm{twist}]}{dt} = k_{0\mathrm{t}} + k_{\mathrm{t}} \frac{\left(\frac{[\mathrm{HIF1}\alpha]}{J_{\mathrm{t}}}\right)^{n_{\mathrm{t}}}}{1 + \left(\frac{[\mathrm{HIF1}\alpha]}{J_{\mathrm{t}}}\right)^{n_{\mathrm{t}}}} + k_{\mathrm{tS}} \frac{\left(\frac{[\mathrm{SNAIL}]}{J_{\mathrm{tS}}}\right)^{n_{\mathrm{tS}}}}{1 + \left(\frac{[\mathrm{SNAIL}]}{J_{\mathrm{tS}}}\right)^{n_{\mathrm{tS}}}} - k_{d\mathrm{t}}[\mathrm{twist}] \tag{S6}$$

$$\frac{d\,[\mathrm{TWIST}]}{dt} = k_{\mathrm{T}} \frac{[\mathrm{twist}]}{1 + \left(\frac{[\mathrm{miR\text{-}129}]}{J_{\mathrm{TR12}}}\right)^{n_{\mathrm{TR12}}}} - k_{d\mathrm{T}}[\mathrm{TWIST}] \tag{S7}$$

$$\frac{d\,[\mathrm{miR\text{-}129}]}{dt} = k_{0\mathrm{R12}} + \frac{k_{\mathrm{R12}}}{1 + \left(\frac{[\mathrm{TWIST}]}{J_{\mathrm{R12T}}}\right)^{n_{\mathrm{R12T}}} + \left(\frac{[\mathrm{SNAIL}]}{J_{\mathrm{R12S}}}\right)^{n_{\mathrm{R12S}}}} - k_{d\mathrm{R12}}[\mathrm{miR\text{-}129}] \tag{S8}$$

$$\frac{d\,[\mathrm{miR\text{-}34}]}{dt} = k_{0\mathrm{R3}} + \frac{k_{\mathrm{R3}}}{1 + \left(\frac{[\mathrm{SNAIL}]}{J_{\mathrm{R3S}}}\right)^{n_{\mathrm{R3S}}} + \left(\frac{[\mathrm{ZEB}]}{J_{\mathrm{R3Z}}}\right)^{n_{\mathrm{R3Z}}}} - k_{d\mathrm{R3}}[\mathrm{miR\text{-}34}] \tag{S9}$$

$$\frac{d\,[\text{miR-200}]}{dt} = k_{0\text{R2}} + \frac{k_{\text{R2}}}{1 + \left(\frac{[\text{ZEB}]}{J_{\text{R2Z}}}\right)^{n_{\text{R2Z}}} + \left(\frac{[\text{SNAIL}]}{J_{\text{R2S}}}\right)^{n_{\text{R2S}}}} - k_{d\text{R2}}[\text{miR-200}] \tag{S10}$$

$$\frac{d\,[\text{N-Cad}]}{dt} = k_{n1}\frac{\left(\frac{[\text{SNAIL}]}{J_{n1}}\right)^{n_1}}{1 + \left(\frac{[\text{SNAIL}]}{J_{n1}}\right)^{n_1}} + k_{n2}\frac{\left(\frac{[\text{ZEB}]}{J_{n2}}\right)^{n_2}}{1 + \left(\frac{[\text{ZEB}]}{J_{n2}}\right)^{n_2}} + k_{n3}\frac{\left(\frac{[\text{TWIST}]}{J_{n3}}\right)^{n_3}}{1 + \left(\frac{[\text{TWIST}]}{J_{n3}}\right)^{n_3}} - k_{dn}[\text{N-Cad}] \tag{S11}$$

$$\frac{d\,[\text{FBP1}]}{dt} = k_{0\text{F}} + \frac{k_{\text{F}}}{1 + \left(\frac{[\text{SNAIL}]}{J_{\text{F}}}\right)^{n_{\text{F}}}} - k_{d\text{F}}[\text{FBP1}] \tag{S12}$$

$$\frac{d\,[\text{GAPDH}]}{dt} = k_{0\text{G}} + \frac{k_{\text{G}}}{1 + \left(\frac{[\text{FBP1}]}{J_{\text{G}}}\right)^{n_{\text{G}}}} - k_{d\text{G}}[\text{GAPDH}] \tag{S13}$$

$$\frac{d\,[\text{HK}]}{dt} = k_{0\text{HK}} + k_{\text{HK}}\left(k_{\text{HK1}}\frac{\left(\frac{[\text{N-Cad}]}{J_{\text{HKN}}}\right)^{n_{\text{HKN}}}}{1 + \left(\frac{[\text{N-Cad}]}{J_{\text{HKN}}}\right)^{n_{\text{HKN}}}} + k_{\text{HK2}}\frac{\left(\frac{[\text{HIF1}\alpha]}{J_{\text{HKH}}}\right)^{n_{\text{HKH}}}}{1 + \left(\frac{[\text{HIF1}\alpha]}{J_{\text{HKH}}}\right)^{n_{\text{HKH}}}} + \frac{k_{\text{HK3}}}{1 + \left(\frac{[\text{FBP1}]}{J_{\text{HKF}}}\right)^{n_{\text{HKF}}}}\right) - k_{d\text{HK}}[\text{HK}] \tag{S14}$$

$$\frac{d\,[\text{LDHA}]}{dt} = k_{0\text{L}} + k_{\text{L}}\left(k_{\text{L1}}\frac{\left(\frac{[\text{HK}]}{J_{\text{LHK}}}\right)^{n_{\text{LHK}}}}{1 + \left(\frac{[\text{HK}]}{J_{\text{LHK}}}\right)^{n_{\text{LHK}}}} + k_{\text{L2}}\frac{\left(\frac{[\text{TWIST}]}{J_{\text{LT}}}\right)^{n_{\text{LT}}}}{1 + \left(\frac{[\text{TWIST}]}{J_{\text{LT}}}\right)^{n_{\text{LT}}}} + k_{\text{L3}}\frac{\left(\frac{[\text{HIF1}\alpha]}{J_{\text{LH}}}\right)^{n_{\text{LH}}}}{1 + \left(\frac{[\text{HIF1}\alpha]}{J_{\text{LH}}}\right)^{n_{\text{LH}}}} + \frac{k_{\text{L4}}}{1 + \left(\frac{[\text{FBP1}]}{J_{\text{LF}}}\right)^{n_{\text{LF}}}}\right) - k_{d\text{L}}[\text{LDHA}] \tag{S15}$$

$$\frac{d\,[\text{E-Cad}]}{dt} = \frac{k_{e1}}{1 + \left(\frac{[\text{SNAIL}]}{J_{e1}}\right)^{n_{e1}}} + \frac{k_{e2}}{1 + \left(\frac{[\text{ZEB}]}{J_{e2}}\right)^{n_{e2}}} + \frac{k_{e3}}{1 + \left(\frac{[\text{TWIST}]}{J_{e3}}\right)^{n_{e3}}} - k_{de}[\text{E-Cad}] \tag{S16}$$

$$\frac{d\,[\text{GLUT}]}{dt} = k_{0\text{GLUT}} + k_{\text{GLUTN}}\frac{\left(\frac{[\text{N-Cad}]}{J_{\text{GLUTN}}}\right)^{n_{\text{GLUTN}}}}{1 + \left(\frac{[\text{N-Cad}]}{J_{\text{GLUTN}}}\right)^{n_{\text{GLUTN}}}} + k_{\text{GLUTH}}\frac{\left(\frac{[\text{HIF1}\alpha]}{J_{\text{GLUTH}}}\right)^{n_{\text{GLUTH}}}}{1 + \left(\frac{[\text{HIF1}\alpha]}{J_{\text{GLUTH}}}\right)^{n_{\text{GLUTH}}}} - k_{d\text{GLUT}}[\text{GLUT}] \tag{S17}$$

$$\frac{d\,[\text{GLC}]}{dt} = \frac{\frac{k_{\text{GLUTmf}}[\text{GLUT}][\text{GLCE}]}{k_{\text{eqGLUT}}} - \frac{k_{\text{GLUTmr}}[\text{GLUT}][\text{GLC}]}{k_{\text{eqGLUT}}}}{1 + \frac{[\text{GLCE}]}{k_{\text{eqGLUT}}} + \frac{[\text{GLC}]}{k_{\text{eqGLUT}}}} - \frac{\frac{k_{\text{HKmf}}[\text{HK}][\text{GLC}]}{k_{\text{eqHKGLC}}} - \frac{k_{\text{HKmr}}[\text{HK}][\text{G6P}]}{k_{\text{eqHKG6P}}}}{1 + \frac{[\text{GLC}]}{k_{\text{eqHKGLC}}} + \frac{[\text{G6P}]}{k_{\text{eqHKG6P}}} + \frac{[\text{GLC}][\text{G6P}]}{k_{\text{eqHKGLC}}k_{\text{eqHKG6Pi}}}} \tag{S18}$$

$$\frac{d\,[\mathrm{G6P}]}{dt} = \frac{\frac{k_{\mathrm{HKmf}}[\mathrm{HK}][\mathrm{GLC}]}{k_{\mathrm{eqHKGLC}}} - \frac{k_{\mathrm{HKmr}}[\mathrm{HK}][\mathrm{G6P}]}{k_{\mathrm{eqHKG6P}}}}{1 + \frac{[\mathrm{GLC}]}{k_{\mathrm{eqHKGLC}}} + \frac{[\mathrm{G6P}]}{k_{\mathrm{eqHKG6P}}} + \frac{[\mathrm{GLC}][\mathrm{G6P}]}{k_{\mathrm{eqHKGLC}}k_{\mathrm{eqHKG6Pi}}}} - \frac{1}{1 + \frac{[\mathrm{FBP1}]}{J_{\mathrm{FBP1}}}} \frac{\frac{[\mathrm{PFK}]}{J_{\mathrm{PFK}}}}{1 + \frac{[\mathrm{PFK}]}{J_{\mathrm{PFK}}}} v_{\mathrm{mfromG6P}} \frac{\left(\frac{[\mathrm{G6P}]}{k_{\mathrm{eqG6P}}}\right)^{n_{\mathrm{G6p}}}}{1 + \left(\frac{[\mathrm{G6P}]}{k_{\mathrm{eqG6P}}}\right)^{n_{\mathrm{G6p}}}} \tag{S19}$$

$$\frac{d\,[\mathrm{PFK}]}{dt} = k_{0\mathrm{PFK}} + \frac{k_{\mathrm{PFK}}}{1 + \frac{k_{\mathrm{PFKFBP}}}{1 + \left(\frac{[\mathrm{F16BP}]}{J_{\mathrm{PFKFBP}}}\right)^{n_{\mathrm{PFKFBP}}}}} + \frac{k_{\mathrm{PFKL}}}{1 + \left(\frac{[\mathrm{LAC}]}{J_{\mathrm{PFKL}}}\right)^{n_{\mathrm{PFKL}}}} - k_{d\mathrm{PFK}}[\mathrm{PFK}] \tag{S20}$$

$$\frac{d\,[\mathrm{F16BP}]}{dt} = r_{\mathrm{atetoF16BP}} \frac{1}{1 + \frac{[\mathrm{FBP1}]}{J_{\mathrm{FBP1}}}} \frac{\frac{[\mathrm{PFK}]}{J_{\mathrm{PFK}}}}{1 + \frac{[\mathrm{PFK}]}{J_{\mathrm{PFK}}}} v_{\mathrm{mfromG6P}} \frac{\left(\frac{[\mathrm{G6P}]}{k_{\mathrm{eqG6P}}}\right)^{n_{\mathrm{G6p}}}}{1 + \left(\frac{[\mathrm{G6P}]}{k_{\mathrm{eqG6P}}}\right)^{n_{\mathrm{G6p}}}} - k_{d\mathrm{F16BP}}[\mathrm{F16BP}] - v_{\mathrm{mfromF16BP}} \frac{\left(\frac{[\mathrm{F16BP}]}{k_{\mathrm{eqFBP}}}\right)^{n_{\mathrm{FBP}}}}{1 + \left(\frac{[\mathrm{F16BP}]}{k_{\mathrm{eqFBP}}}\right)^{n_{\mathrm{FBP}}}} \tag{S21}$$

$$\frac{d\,[\mathrm{G3P}]}{dt} = r_{\mathrm{atetoG3P}} v_{\mathrm{mfromF16BP}} \frac{\left(\frac{[\mathrm{F16BP}]}{k_{\mathrm{eqFBP}}}\right)^{n_{\mathrm{FBP}}}}{1 + \left(\frac{[\mathrm{F16BP}]}{k_{\mathrm{eqFBP}}}\right)^{n_{\mathrm{FBP}}}} - k_{d\mathrm{G3P}}[\mathrm{G3P}] - \frac{\frac{[\mathrm{GAPDH}]}{J_{\mathrm{GAPDH}}}}{1 + \frac{[\mathrm{GAPDH}]}{J_{\mathrm{GAPDH}}}} v_{\mathrm{mfromG3P}} \frac{\left(\frac{[\mathrm{G3P}]}{k_{\mathrm{eqG3P}}}\right)^{n_{\mathrm{G3P}}}}{1 + \left(\frac{[\mathrm{G3P}]}{k_{\mathrm{eqG3P}}}\right)^{n_{\mathrm{G3P}}}} \tag{S22}$$

$$\frac{d\,[\mathrm{PYRc}]}{dt} = \frac{\frac{[\mathrm{GAPDH}]}{J_{\mathrm{GAPDH}}}}{1 + \frac{[\mathrm{GAPDH}]}{J_{\mathrm{GAPDH}}}} v_{\mathrm{mfromG3P}} \frac{\left(\frac{[\mathrm{G3P}]}{k_{\mathrm{eqG3P}}}\right)^{n_{\mathrm{G3P}}}}{1 + \left(\frac{[\mathrm{G3P}]}{k_{\mathrm{eqG3P}}}\right)^{n_{\mathrm{G3P}}}} - k_{d\mathrm{PYR}}[\mathrm{PYRc}] - \frac{\frac{k_{\mathrm{Lmf}}[\mathrm{LDHA}][\mathrm{PYRc}]}{k_{\mathrm{eqLPYR}}} - \frac{k_{\mathrm{Lmr}}[\mathrm{LDHA}][\mathrm{LAC}]}{k_{\mathrm{eqLLAC}}}}{1 + \frac{[\mathrm{PYRc}]}{k_{\mathrm{eqLPYR}}} + \frac{[\mathrm{LAC}]}{k_{\mathrm{eqLLAC}}} + \frac{[\mathrm{PYRc}][\mathrm{LAC}]}{k_{\mathrm{eqLPYR}}k_{\mathrm{eqLLACi}}}} - \frac{k_{\mathrm{PYRtrans}}[\mathrm{PYRc}]}{1 + \frac{[\mathrm{PYRm}]}{J_{\mathrm{PYRm}}}} \tag{S23}$$

$$\frac{d\,[\mathrm{LAC}]}{dt} = \frac{\frac{k_{\mathrm{Lmf}}[\mathrm{LDHA}][\mathrm{PYRc}]}{k_{\mathrm{eqLPYR}}} - \frac{k_{\mathrm{Lmr}}[\mathrm{LDHA}][\mathrm{LAC}]}{k_{\mathrm{eqLLAC}}}}{1 + \frac{[\mathrm{PYRc}]}{k_{\mathrm{eqLPYR}}} + \frac{[\mathrm{LAC}]}{k_{\mathrm{eqLLAC}}} + \frac{[\mathrm{PYRc}][\mathrm{LAC}]}{k_{\mathrm{eqLPYR}}k_{\mathrm{eqLLACi}}}} - k_{\mathrm{LACout}}[\mathrm{LAC}] + k_{\mathrm{LACin}}[\mathrm{LACe}] \tag{S24}$$

$$\frac{d\,[\mathrm{LACe}]}{dt} = k_{\mathrm{solvent}}(k_{\mathrm{LACout}}[\mathrm{LAC}] - k_{\mathrm{LACin}}[\mathrm{LACe}]) - k_{d\mathrm{LACe}}[\mathrm{LACe}] \tag{S25}$$

$$\frac{d\,[\mathrm{PYRm}]}{dt} = \frac{k_{\mathrm{PYRtrans}}[\mathrm{PYRc}]}{1 + \frac{[\mathrm{PYRm}]}{J_{\mathrm{PYRm}}}} - k_{\mathrm{PYRconsume}}[\mathrm{PYRm}] \frac{\left(\frac{[\mathrm{O_2}]}{J_{\mathrm{O2}}}\right)^{n_{\mathrm{O2}}}}{1 + \left(\frac{[\mathrm{O_2}]}{J_{\mathrm{O2}}}\right)^{n_{\mathrm{O2}}}} - k_{d\mathrm{PYRm}}[\mathrm{PYRm}] \tag{S26}$$

S2 Model variables and baseline settings

The state variables and initial conditions are listed in Table S1, and the nominal kinetic parameters are listed in Table S2. Unless otherwise stated, time is measured in hours (h), concentrations are measured in micromolar (μM), and reaction rates are measured in μM/h. Although membrane proteins such as E-cadherin and N-cadherin are physically associated with local surface densities, the present model is spatially lumped; therefore, these proteins are represented by equivalent volumetric concentrations that describe whole-cell relative abundance.

Table S1: State variables and initial conditions

**Variable**	**Description**	**Initial value**
[HIF1α]	HIF-1α protein concentration	0.11
[snail]	SNAIL transcription factor mRNA concentration	0.02
[SNAIL]	SNAIL protein concentration	0.02
[zeb]	ZEB transcription factor mRNA concentration	0.03
[ZEB]	ZEB protein concentration	0.01
[twist]	TWIST transcription factor mRNA concentration	0.015
[TWIST]	TWIST protein concentration	0.005
[miR-129]	microRNA-129 concentration	0.27
[miR-34]	microRNA-34 concentration	0.3
[miR-200]	microRNA-200 concentration	0.35
[E-Cad]	Epithelial marker E-cadherin protein concentration	3.6
[N-Cad]	Mesenchymal marker N-cadherin protein concentration	0.1
[FBP1]	Fructose-1,6-bisphosphatase concentration	0.7
[GAPDH]	Glyceraldehyde-3-phosphate dehydrogenase protein concentration	0.4
[HK]	Hexokinase protein concentration	0.9
[LDHA]	Lactate dehydrogenase A protein concentration	0.3
[GLUT]	Glucose transporter protein (GLUT1) concentration	0.3
[GLC]	Intracellular glucose concentration	0.1
[G6P]	Glucose-6-phosphate concentration	0.1
[PFK]	Phosphofructokinase (PFK-1) concentration	0.1
[F16BP]	Fructose-1,6-bisphosphate concentration	0.15
[G3P]	Glyceraldehyde-3-phosphate concentration	0.1
[PYRc]	Cytosolic pyruvate concentration	0.01
[LAC]	Intracellular lactate concentration	0.01
[LACe]	Extracellular microenvironmental lactate concentration	0.02
[PYRm]	Mitochondrial pyruvate concentration	0.01

Table S2: Baseline parameter settings for the coupled EMT–glycolysis model

**Parameter**	**Description**	**Nominal value**	**Source or rationale**
External inputs			
O_2	Oxygen partial-pressure signal	0.1	Assumed
GLCE	Extracellular glucose concentration	10	Assumed
HIF-1α module			
$k_{0\mathrm{H}}$	Basal synthesis rate of HIF-1α	1.5	Assumed
$k_{d0\mathrm{H}}$	Basal oxygen-independent degradation rate constant of HIF-1α	0.4	[82]
$k_{d\mathrm{H}}$	Maximal oxygen-dependent degradation rate constant of HIF-1α	4.5	[83]
J_{H}	Michaelis-like constant for oxygen-promoted HIF-1α degradation	8	Assumed
n_{H}	Hill coefficient for oxygen-promoted HIF-1α degradation	2	Assumed
J_{HLAC}	Michaelis-like constant for lactate-mediated inhibition of HIF-1α degradation	0.06	Assumed
n_{HLAC}	Hill coefficient for lactate-mediated inhibition of HIF-1α degradation	2	Assumed
SNAIL mRNA module			
$k_{0\mathrm{s}}$	Basal synthesis rate of SNAIL mRNA	0.0002	Assumed
k_{s}	Transcriptional rate coefficient for SNAIL mRNA	0.3	[17]
k_{s1}	Induction coefficient for TWIST-promoted SNAIL transcription	0.05	Assumed
J_{sT}	Michaelis-like constant for TWIST-promoted SNAIL transcription	0.13	Assumed
n_{sT}	Hill coefficient for TWIST-promoted SNAIL transcription	2	Assumed
k_{s2}	Induction coefficient for HIF-1α-promoted SNAIL transcription	0.5	Assumed
J_{sH}	Michaelis-like constant for HIF-1α-promoted SNAIL transcription	1.25	Assumed
n_{sH}	Hill coefficient for HIF-1α-promoted SNAIL transcription	2	Assumed
k_{s3}	Induction coefficient for GAPDH-promoted SNAIL transcription	0.05	Assumed
J_{sG}	Michaelis-like constant for GAPDH-promoted SNAIL transcription	0.8	Assumed
n_{sG}	Hill coefficient for GAPDH-promoted SNAIL transcription	2	Assumed
$k_{d\mathrm{s}}$	Degradation rate constant of SNAIL mRNA	1.5	[84]
SNAIL protein module			
k_{S}	Maximal translation rate constant of SNAIL protein	18	[15,17]
J_{SR3}	Michaelis-like constant for miR-34-mediated inhibition of SNAIL translation	0.09	[15,17]
n_{SR3}	Hill coefficient for miR-34-mediated inhibition of SNAIL translation	2	Assumed
$k_{d\mathrm{S}}$	Degradation rate constant of SNAIL protein	1.66	[85]
ZEB mRNA module			
$k_{0\mathrm{z}}$	Basal synthesis rate of ZEB mRNA	0.003	[15]
k_{z}	Signal-induced synthesis rate coefficient of ZEB mRNA	0.06	[15]
k_{z1}	Induction coefficient for SNAIL-promoted ZEB transcription	0.16	Assumed
J_{zS}	Michaelis-like constant for SNAIL-promoted ZEB transcription	0.65	Assumed
n_{zS}	Hill coefficient for SNAIL-promoted ZEB transcription	2	Assumed
k_{z2}	Induction coefficient for LDHA-promoted ZEB transcription	0.2	Assumed
J_{zL}	Michaelis-like constant for LDHA-promoted ZEB transcription	0.8	Assumed
n_{zL}	Hill coefficient for LDHA-promoted ZEB transcription	2	Assumed
$k_{d\mathrm{z}}$	Degradation rate constant of ZEB mRNA	0.09	[15]
ZEB protein module			
k_{Z}	Maximal translation rate constant of ZEB protein	17	[15]

Continued on next page

Table S2 continued from previous page

Parameter	Description	Nominal value	Source or rationale
J_{ZR2}	Michaelis-like constant for miR-200-mediated inhibition of ZEB translation	0.06	[15]
n_{ZR2}	Hill coefficient for miR-200-mediated inhibition of ZEB translation	2	Assumed
$k_{d\mathrm{Z}}$	Degradation rate constant of ZEB protein	1.66	[15]
TWIST mRNA module			
$k_{0\mathrm{t}}$	Basal synthesis rate of TWIST mRNA	0.0002	[17]
k_{t}	Maximal rate of HIF-1α-promoted TWIST transcription	0.038	[17]
J_{t}	Michaelis-like constant for HIF-1α-promoted TWIST transcription	1.4	Assumed
n_{t}	Hill coefficient for HIF-1α-promoted TWIST transcription	2	Assumed
k_{tS}	Maximal rate of SNAIL-promoted TWIST transcription	0.01	Assumed
J_{tS}	Michaelis-like constant for SNAIL-promoted TWIST transcription	0.7	Assumed
n_{tS}	Hill coefficient for SNAIL-promoted TWIST transcription	2	Assumed
$k_{d\mathrm{t}}$	Degradation rate constant of TWIST mRNA	0.14	[86]
TWIST protein module			
k_{T}	Maximal translation rate constant of TWIST protein	1.8	[17]
J_{TR12}	Michaelis-like constant for miR-129-mediated inhibition of TWIST translation	0.07	[17]
n_{TR12}	Hill coefficient for miR-129-mediated inhibition of TWIST translation	2	Assumed
$k_{d\mathrm{T}}$	Degradation rate constant of TWIST protein	0.35	[87]
microRNA modules			
$k_{0\mathrm{R12}}$	Basal synthesis rate of miR-129	0.02	[17]
k_{R12}	Maximal induced synthesis rate of miR-129	0.17	[17]
J_{R12T}	Michaelis-like constant for TWIST-mediated repression of miR-129 synthesis	0.2	Assumed
n_{R12T}	Hill coefficient for TWIST-mediated repression of miR-129 synthesis	2	Assumed
J_{R12S}	Michaelis-like constant for SNAIL-mediated repression of miR-129 synthesis	3.5	[17]
n_{R12S}	Hill coefficient for SNAIL-mediated repression of miR-129 synthesis	2	Assumed
$k_{d\mathrm{R12}}$	Degradation rate constant of miR-129	0.7	[17]
$k_{0\mathrm{R3}}$	Basal synthesis rate of miR-34	0.014	[17]
k_{R3}	Maximal induced synthesis rate of miR-34	0.072	[17]
J_{R3S}	Michaelis-like constant for SNAIL-mediated repression of miR-34 synthesis	0.11	Assumed
n_{R3S}	Hill coefficient for SNAIL-mediated repression of miR-34 synthesis	2	Assumed
J_{R3Z}	Michaelis-like constant for ZEB-mediated repression of miR-34 synthesis	0.36	[15]
n_{R3Z}	Hill coefficient for ZEB-mediated repression of miR-34 synthesis	2	Assumed
$k_{d\mathrm{R3}}$	Degradation rate constant of miR-34	0.28	[88]
$k_{0\mathrm{R2}}$	Basal synthesis rate of miR-200	0.002	[15]
k_{R2}	Maximal induced synthesis rate of miR-200	0.12	Assumed
J_{R2Z}	Michaelis-like constant for ZEB-mediated repression of miR-200 synthesis	0.25	[15]
n_{R2Z}	Hill coefficient for ZEB-mediated repression of miR-200 synthesis	2	Assumed
J_{R2S}	Michaelis-like constant for SNAIL-mediated repression of miR-200 synthesis	0.6	Assumed
n_{R2S}	Hill coefficient for SNAIL-mediated repression of miR-200 synthesis	2	Assumed
$k_{d\mathrm{R2}}$	Degradation rate constant of miR-200	0.35	[89]
E-cadherin module			
k_{e1}	Maximal E-cadherin expression rate for the SNAIL-regulated term	0.7	Assumed
J_{e1}	Michaelis-like constant for SNAIL-mediated repression of E-cadherin expression	0.2	[15]
n_{e1}	Hill coefficient for SNAIL-mediated repression of E-cadherin expression	2	Assumed

Continued on next page

Table S2 continued from previous page

Parameter	Description	Nominal value	Source or rationale
k_{e2}	Maximal E-cadherin expression rate for the ZEB-regulated term	0.5	Assumed
J_{e2}	Michaelis-like constant for ZEB-mediated repression of E-cadherin expression	0.4	Assumed
n_{e2}	Hill coefficient for ZEB-mediated repression of E-cadherin expression	2	Assumed
k_{e3}	Maximal E-cadherin expression rate for the TWIST-regulated term	0.6	Assumed
J_{e3}	Michaelis-like constant for TWIST-mediated repression of E-cadherin expression	0.4	Assumed
n_{e3}	Hill coefficient for TWIST-mediated repression of E-cadherin expression	2	Assumed
k_{de}	Degradation rate constant of E-cadherin	0.5	[15,17]
N-cadherin module			
k_{n1}	Maximal SNAIL-induced expression rate of N-cadherin	0.7	Assumed
J_{n1}	Michaelis-like constant for SNAIL-induced N-cadherin expression	0.2	[15]
n_1	Hill coefficient for SNAIL-induced N-cadherin expression	2	Assumed
k_{n2}	Maximal ZEB-induced expression rate of N-cadherin	0.5	Assumed
J_{n2}	Michaelis-like constant for ZEB-induced N-cadherin expression	0.4	Assumed
n_2	Hill coefficient for ZEB-induced N-cadherin expression	2	Assumed
k_{n3}	Maximal TWIST-induced expression rate of N-cadherin	0.6	Assumed
J_{n3}	Michaelis-like constant for TWIST-induced N-cadherin expression	0.4	Assumed
n_3	Hill coefficient for TWIST-induced N-cadherin expression	2	Assumed
k_{dn}	Degradation rate constant of N-cadherin protein	0.5	[15]
Glycolytic inhibitor and GAPDH modules			
$k_{0\mathrm{F}}$	Basal synthesis rate of FBP1	0.2	Assumed
k_{F}	Maximal induced synthesis rate of FBP1	0.5	Assumed
J_{F}	Michaelis-like constant for SNAIL-mediated repression of FBP1	0.4	Assumed
n_{F}	Hill coefficient for SNAIL-mediated repression of FBP1	2	Assumed
$k_{d\mathrm{F}}$	Degradation rate constant of FBP1	1	[90,91]
$k_{0\mathrm{G}}$	Basal synthesis rate of GAPDH	0.1	Assumed
k_{G}	Maximal induced synthesis rate of GAPDH	1	Assumed
J_{G}	Michaelis-like constant for FBP1-mediated repression of GAPDH	0.5	Assumed
n_{G}	Hill coefficient for FBP1-mediated repression of GAPDH	2	Assumed
$k_{d\mathrm{G}}$	Degradation rate constant of GAPDH	1	[92]
Hexokinase module			
$k_{0\mathrm{HK}}$	Basal synthesis rate of hexokinase	0.9	Assumed
k_{HK}	Maximal multiplier for signal-dependent activation of hexokinase	0.2	Assumed
k_{HK1}	Relative induction coefficient for N-cadherin-mediated activation of hexokinase	1	Assumed
J_{HKN}	Michaelis-like constant for N-cadherin-mediated activation of hexokinase	1.5	Assumed
n_{HKN}	Hill coefficient for N-cadherin-mediated activation of hexokinase	2	Assumed
k_{HK2}	Relative induction coefficient for HIF-1α-mediated activation of hexokinase	1	Assumed
J_{HKH}	Michaelis-like constant for HIF-1α-mediated activation of hexokinase	2	Assumed
n_{HKH}	Hill coefficient for HIF-1α-mediated activation of hexokinase	2	Assumed
k_{HK3}	Regulatory coefficient for FBP1-mediated inhibition of hexokinase	1	Assumed
J_{HKF}	Michaelis-like constant for FBP1-mediated inhibition of hexokinase	0.3	Assumed
n_{HKF}	Hill coefficient for FBP1-mediated inhibition of hexokinase	2	Assumed
$k_{d\mathrm{HK}}$	Degradation rate constant of hexokinase	1	Assumed
LDHA module			

Continued on next page

Table S2 continued from previous page

Parameter	Description	Nominal value	Source or rationale
$k_{0\mathrm{L}}$	Basal synthesis rate of lactate dehydrogenase A	0.1	Assumed
k_{L}	Maximal multiplier for signal-dependent activation of LDHA	0.5	Assumed
k_{L1}	Relative induction coefficient for HK-mediated activation of LDHA	1	Assumed
J_{LHK}	Michaelis-like constant for HK-mediated activation of LDHA	2	Assumed
n_{LHK}	Hill coefficient for HK-mediated activation of LDHA	2	Assumed
k_{L2}	Relative induction coefficient for TWIST-mediated activation of LDHA	2	Assumed
J_{LT}	Michaelis-like constant for TWIST-mediated activation of LDHA	0.4	Assumed
n_{LT}	Hill coefficient for TWIST-mediated activation of LDHA	2	Assumed
k_{L3}	Relative induction coefficient for HIF-1α-mediated activation of LDHA	1	Assumed
J_{LH}	Michaelis-like constant for HIF-1α-mediated activation of LDHA	2	Assumed
n_{LH}	Hill coefficient for HIF-1α-mediated activation of LDHA	2	Assumed
k_{L4}	Regulatory coefficient for FBP1-mediated inhibition of LDHA	1	Assumed
J_{LF}	Michaelis-like constant for FBP1-mediated inhibition of LDHA	0.3	Assumed
n_{LF}	Hill coefficient for FBP1-mediated inhibition of LDHA	2	Assumed
$k_{d\mathrm{L}}$	Degradation rate constant of LDHA	1	Assumed
GLUT module			
$k_{0\mathrm{GLUT}}$	Basal synthesis rate of GLUT	1	Assumed
k_{GLUTN}	Maximal rate of N-cadherin-mediated activation of GLUT	0.2	Assumed
J_{GLUTN}	Michaelis-like constant for N-cadherin-mediated activation of GLUT	2	Assumed
n_{GLUTN}	Hill coefficient for N-cadherin-mediated activation of GLUT	2	Assumed
k_{GLUTH}	Maximal rate of HIF-1α-mediated activation of GLUT	0.5	Assumed
J_{GLUTH}	Michaelis-like constant for HIF-1α-mediated activation of GLUT	2	Assumed
n_{GLUTH}	Hill coefficient for HIF-1α-mediated activation of GLUT	2	Assumed
$k_{d\mathrm{GLUT}}$	Degradation rate constant of GLUT	1	[93]
PFK module			
$k_{0\mathrm{PFK}}$	Basal synthesis rate of PFK	0.02	Assumed
k_{PFK}	Maximal activation/synthesis rate of PFK	6	Assumed
k_{PFKFBP}	Multiplier for allosteric activation of PFK by fructose-1,6-bisphosphate	100	Assumed
J_{PFKFBP}	Michaelis-like constant for F16BP-mediated activation of PFK	0.2	Assumed
n_{PFKFBP}	Hill coefficient for F16BP-mediated activation of PFK	4	Assumed
k_{PFKL}	Maximal rate for lactate-regulated PFK production	0.1	Assumed
J_{PFKL}	Michaelis-like constant for lactate regulation of PFK	0.2	Assumed
n_{PFKL}	Hill coefficient for lactate regulation of PFK	2	Assumed
$k_{d\mathrm{PFK}}$	Degradation rate constant of PFK	5	Assumed
Metabolic flux constants: GLUT/HK			
k_{GLUTmf}	Forward glucose transport rate constant through GLUT	10	[69]
k_{GLUTmr}	Reverse glucose transport rate constant through GLUT	7.67	[20,69]
k_{eqGLUT}	Equilibrium constant for glucose transmembrane transport	1	Assumed
k_{HKmf}	Forward catalytic rate constant of HK	25	Assumed
k_{HKmr}	Reverse catalytic rate constant of HK	5	[69]
k_{eqHKGLC}	Equilibrium constant for HK binding to glucose	0.1	[20,69]
k_{eqHKG6P}	Equilibrium constant for HK binding to glucose-6-phosphate	0.047	[20,69]
k_{eqHKG6Pi}	Internal inhibition constant of the HK catalytic complex	0.047	[20,69]

Continued on next page

Table S2 continued from previous page

**Parameter**	**Description**	**Nominal value**	**Source or rationale**
Metabolic flux constants: G6P/F16BP			
$r_{\mathrm{atetoF16BP}}$	Partition ratio for conversion of G6P to fructose-1,6-bisphosphate	0.9	Assumed
J_{FBP1}	Michaelis-like constant for FBP1-mediated inhibition of metabolic flux	2.4	Assumed
J_{PFK}	Michaelis-like constant for saturating PFK catalysis	1	Assumed
v_{mfromG6P}	Maximal downstream metabolic flux from G6P	5	Assumed
k_{eqG6P}	Equilibrium constant for the G6P metabolic reaction	0.21	[94]
n_{G6p}	Hill coefficient for the G6P metabolic reaction	2	Assumed
$k_{d\mathrm{F16BP}}$	Basal consumption rate constant of F16BP	2.55	Assumed
Metabolic flux constants: G3P			
r_{atetoG3P}	Partition ratio for conversion of F16BP to glyceraldehyde-3-phosphate	1.8	Assumed
$v_{\mathrm{mfromF16BP}}$	Maximal downstream conversion rate from F16BP	3	Assumed
k_{eqFBP}	Equilibrium constant for the F16BP metabolic reaction	1	Assumed
n_{FBP}	Hill coefficient for the F16BP metabolic reaction	2	Assumed
$k_{d\mathrm{G3P}}$	Basal consumption rate constant of G3P	2	Assumed
Metabolic flux constants: pyruvate/GAPDH			
J_{GAPDH}	Michaelis-like saturation constant for GAPDH-catalyzed flux	0.1	Assumed
v_{mfromG3P}	Maximal conversion rate from G3P to pyruvate	8	[94,95]
k_{eqG3P}	Equilibrium constant for the G3P conversion reaction	1.7	[94,95]
n_{G3P}	Hill coefficient for the G3P conversion reaction	2	Assumed
$k_{d\mathrm{PYR}}$	Basal nonspecific consumption rate constant of cytosolic pyruvate	0.05	Assumed
Metabolic flux constants: LDH/lactate			
k_{Lmf}	Forward catalytic rate constant for LDHA-mediated lactate production	0.45	Assumed
k_{Lmr}	Reverse catalytic rate constant for LDHA-mediated lactate consumption	2.5	Assumed
k_{eqLPYR}	Equilibrium constant for LDHA binding to pyruvate	1.37	[20,69]
k_{eqLLAC}	Equilibrium constant for LDHA binding to lactate	5	[69]
k_{eqLLACi}	Internal inhibition constant of the LDHA catalytic complex	7.33	[20,69]
k_{solvent}	Diffusion and dilution coefficient for intracellular–extracellular lactate exchange	2	Assumed
k_{LACout}	Rate constant for lactate efflux from the cell	30	Assumed
k_{LACin}	Rate constant for extracellular lactate uptake into the cell	10	Assumed
$k_{d\mathrm{LACe}}$	Basal loss rate constant of extracellular lactate	0.5	Assumed
Metabolic flux constants: mitochondrial pyruvate			
k_{PYRtrans}	Maximal rate of pyruvate transport into mitochondria	0.1	Assumed
J_{PYRm}	Michaelis-like constant for mitochondrial pyruvate uptake	0.5	Assumed
$k_{\mathrm{PYRconsume}}$	Maximal rate of pyruvate consumption by the mitochondrial TCA cycle	10	Assumed
J_{O2}	Michaelis-like constant for oxygen-promoted mitochondrial pyruvate consumption	3	Assumed
n_{O2}	Hill coefficient for oxygen-promoted mitochondrial pyruvate consumption	2	Assumed
$k_{d\mathrm{PYRm}}$	Basal consumption rate constant of mitochondrial pyruvate	0.005	Assumed

S3 Parameter sensitivity analysis

In this subsection, we present a comprehensive parameter sensitivity analysis to evaluate the robustness of the EMT–glycolysis mathematical model. The layout below details the computational protocol and reports the ranked sensitivity results, highlighting the regulatory hierarchy of the network.

S3.1 Analysis objective

The primary objective of the sensitivity analysis is to quantify how perturbations of kinetic and regulatory parameters affect the steady-state outputs of the model. We specifically focused on output variables that define the phenotypic state of the cell, starting with the core epithelial marker, E-cadherin (E-Cad).

S3.2 Perturbation protocol

Each model parameter p_i was perturbed individually around its nominal value p_i^0 while all other parameters were held constant. To evaluate the local robustness of the system, a symmetric perturbation range of 10% was applied:

$$p_i^- = p_i^0(1 - 0.1), \qquad p_i^+ = p_i^0(1 + 0.1). \tag{S27}$$

For parameters constrained by biological physics to be strictly positive, perturbations were strictly bounded to avoid nonphysical negative values.

S3.3 Sensitivity coefficient

To standardize the comparison across parameters with varying magnitudes, the normalized finite-difference sensitivity coefficient for the output metric Y_j (e.g., the steady-state concentration of E-Cad) with respect to parameter p_i is defined as:

$$S_{ij} = \frac{p_i^0}{Y_j^0} \frac{Y_j(p_i^+) - Y_j(p_i^-)}{p_i^+ - p_i^-}, \tag{S28}$$

where Y_j^0 represents the unperturbed steady-state output. This formulation effectively captures the relative change in the output corresponding to the applied perturbation.

S3.4 Global sensitivity of E-cadherin expression

To visualize the global regulatory impact on the epithelial phenotype, we ranked the parameters based on their influence on the steady-state concentration of E-cadherin. As shown in Figure S1, the results are presented as a tornado plot displaying both the top 70 most sensitive parameters (left panel) and the bottom 70 least sensitive parameters (right panel) in response to a ±10% perturbation.

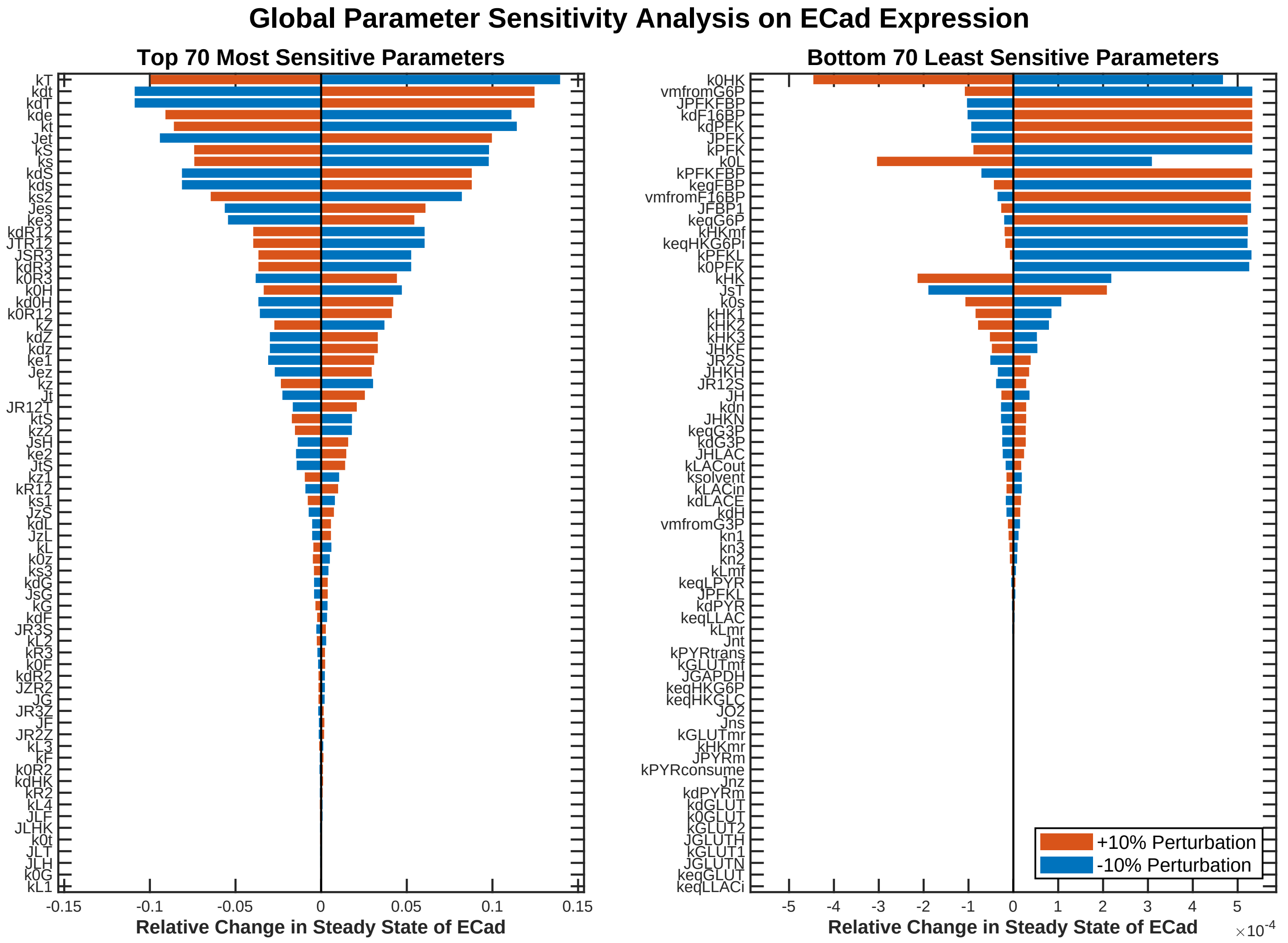

Figure S1: Global parameter sensitivity analysis on the steady-state expression of E-cadherin. Red bars represent a +10% increase in the parameter value, while blue bars represent a −10% decrease. The left panel shows the top 70 most influential parameters, whereas the right panel displays the 70 parameters with the least impact on E-Cad levels.

The analysis reveals a distinct regulatory hierarchy. The top-ranked parameters (Figure S1, left panel) are predominantly kinetic constants associated with the core EMT transcriptional circuits, such as kT, kS, and cadherin synthesis and degradation rates ($ke3$, kde). Perturbation of these upstream regulators induces substantial relative changes in E-cadherin expression, reaching up to a 15% deviation from the baseline. This confirms that the canonical SNAIL/TWIST/ZEB axes firmly govern the EMT status.

Conversely, while the majority of metabolic constants—including glycolytic enzyme production rates, glycolytic reaction rates, and regulatory thresholds (e.g., $k0HK$, $vmfromG6P$, $JPFKFBP$)—cluster among the bottom-ranked parameters with negligible relative changes in E-cadherin (hovering at an order of magnitude of 10^{-4}), this downstream decoupling is not absolute. Notably, specific metabolic control points exhibit a markedly more pronounced influence on the epithelial status. For instance, parameters explicitly coupling the metabolic and transcriptional modules—such as the rate constant for the LDHA-mediated transcription of ZEB mRNA ($kz2$) and the LDHA degradation rate (kdL)—demonstrate a significant capability to alter E-cadherin levels, inducing variations of several percent. This nuanced contrast indicates that although bulk fluctuations in general glycolytic fluxes exert minimal retrograde influence on EMT, targeted metabolic nodes can still provide crucial feedback to drive or modulate the phenotypic transition.

S3.5 Global sensitivity at critical bifurcation points

In addition to the steady-state analysis, it is crucial to understand how parameter perturbations shift the threshold of phenotypic transitions. To this end, we performed a global parameter sensitivity analysis at the critical saddle-node (SN) bifurcation points. As depicted in Figure 2B of the main text, the system exhibits multiple SN bifurcations in response to varying oxygen levels.

Specifically, SN6, SN4, and SN2 correspond to the thresholds for the sequential activation of the EMT program as oxygen levels decrease (hypoxia). Conversely, SN5, SN3, and SN1 represent the stepwise mesenchymal-to-epithelial transition (MET) switches triggered as oxygen levels recover. Furthermore, we evaluated the bifurcation points that govern the metabolic reprogramming: the triggering of the glycolysis switch during hypoxia (Glycolysis ON) and its subsequent deactivation during reoxygenation (Glycolysis OFF). These complementary analyses highlight the distinct parameter sets that dictate the activation barriers for EMT and its robust maintenance through glycolytic metabolism.

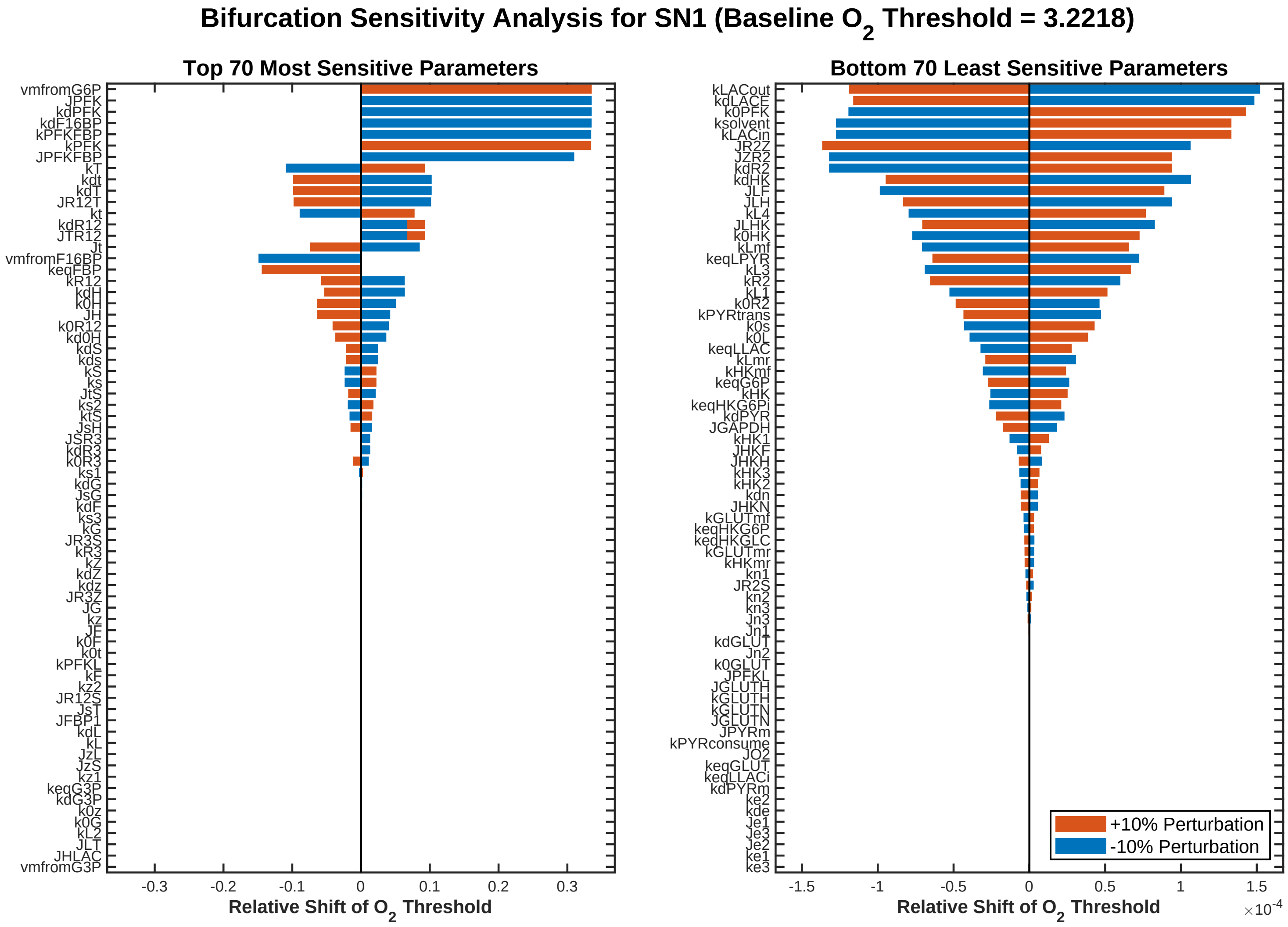

Figure S2: Parameter sensitivity analysis at the saddle-node bifurcation point SN1. This point represents a stepwise MET switch triggered as oxygen levels increase.

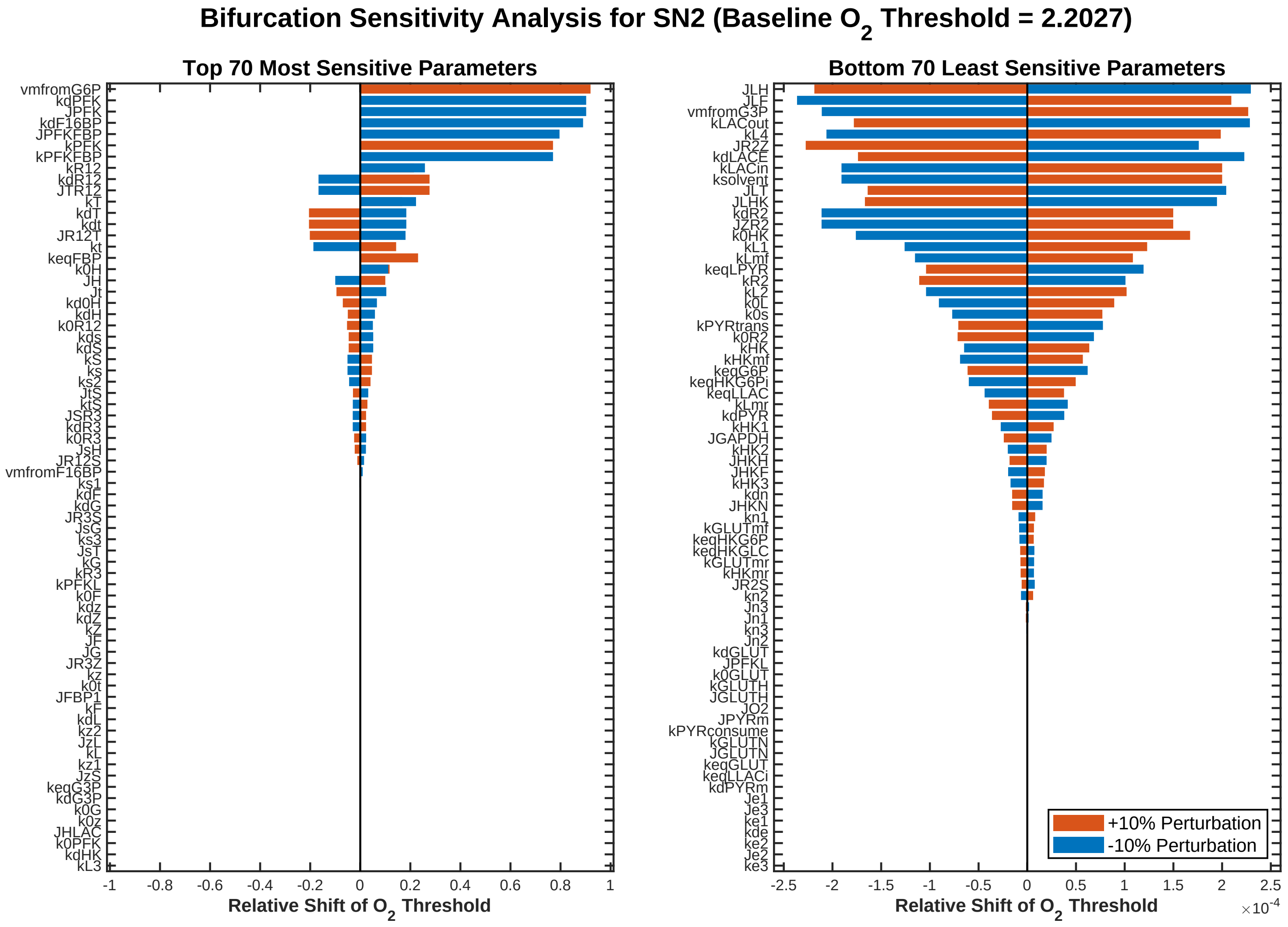

Figure S3: Parameter sensitivity analysis at the saddle-node bifurcation point SN2. This point corresponds to a sequential EMT switch activated as oxygen levels decrease.

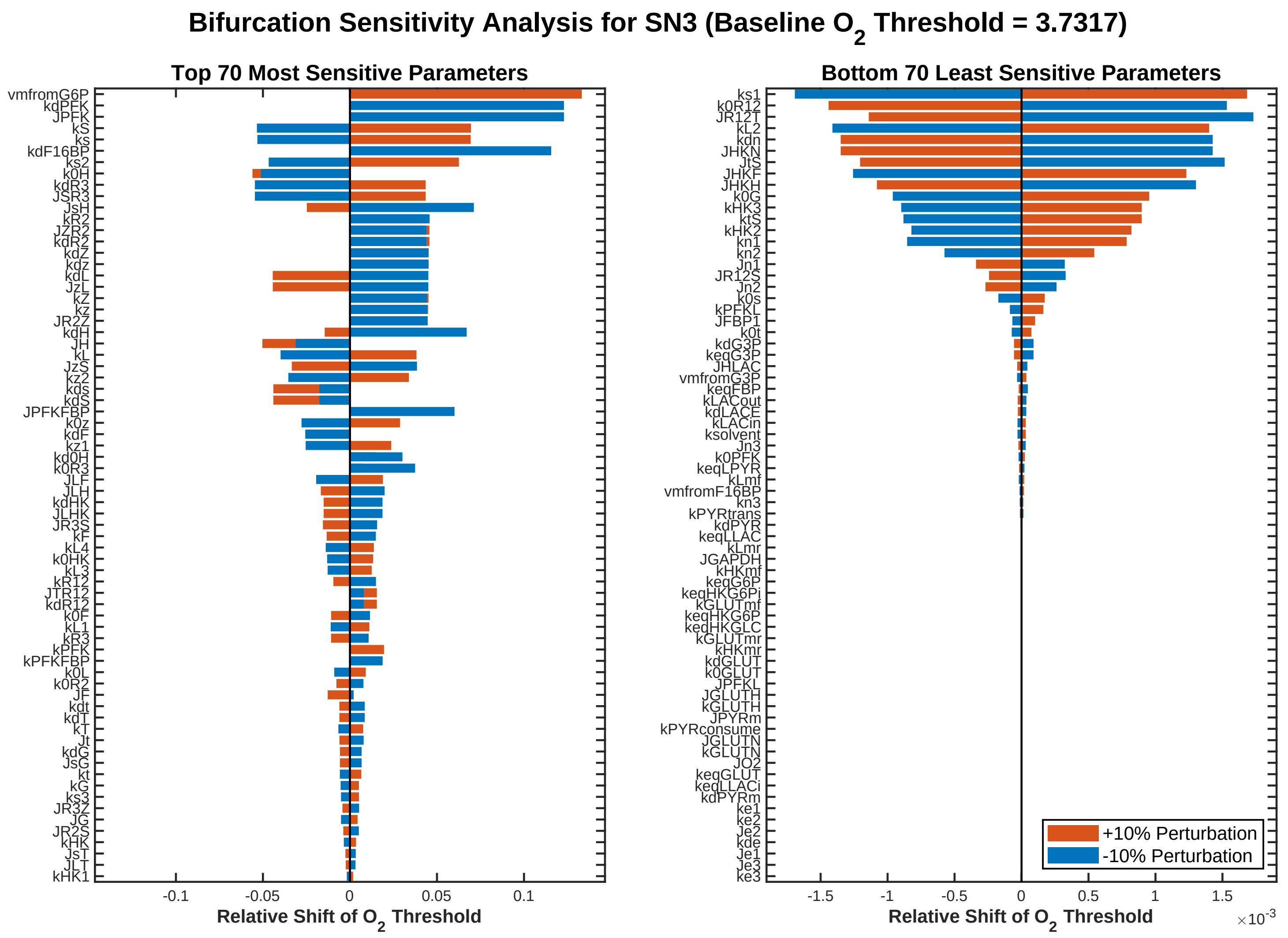

Figure S4: Parameter sensitivity analysis at the saddle-node bifurcation point SN3. This point represents a stepwise MET switch triggered as oxygen levels increase.

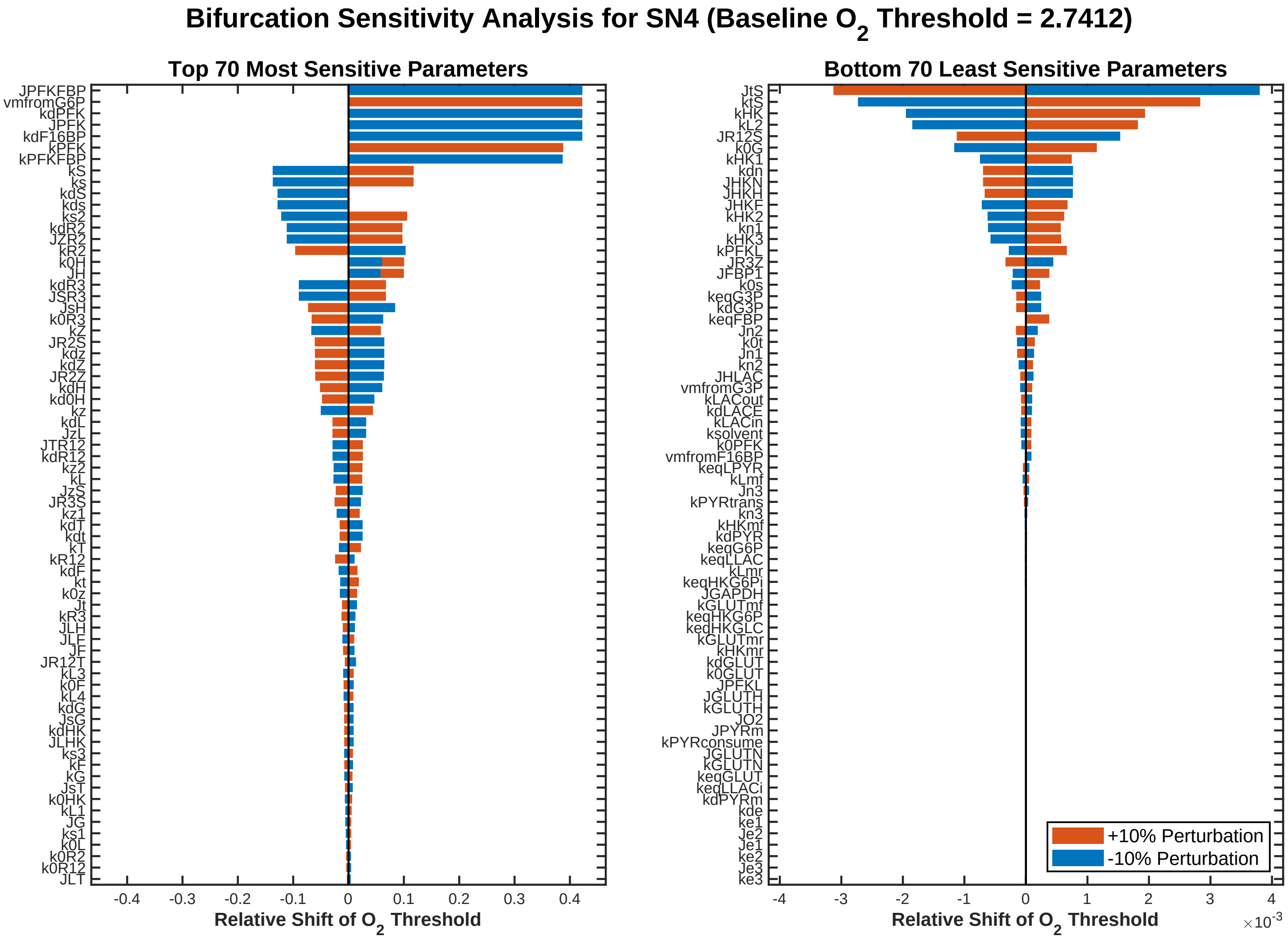

Figure S5: Parameter sensitivity analysis at the saddle-node bifurcation point SN4. This point corresponds to a sequential EMT switch activated as oxygen levels decrease.

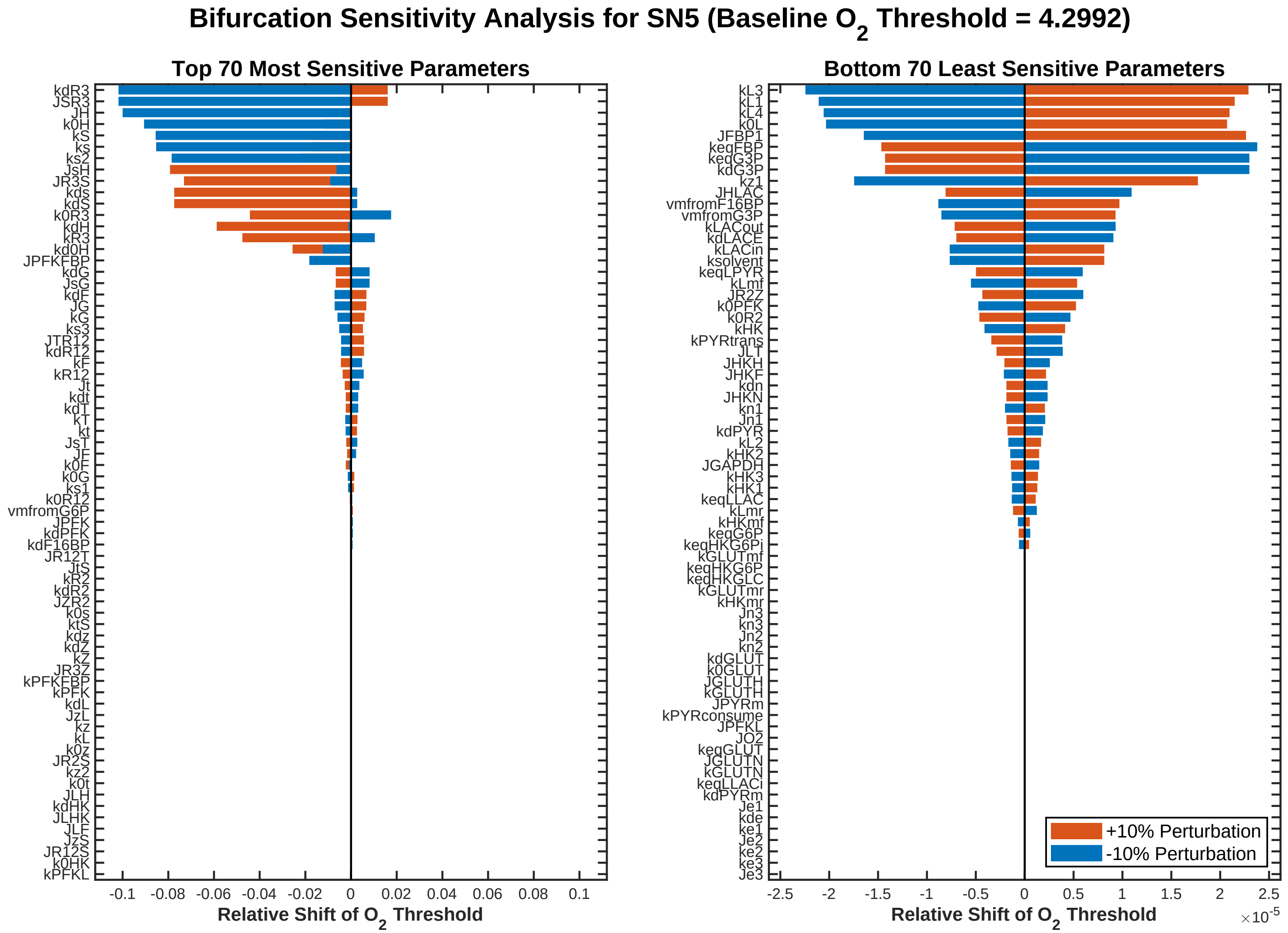

Figure S6: Parameter sensitivity analysis at the saddle-node bifurcation point SN5. This point represents a stepwise MET switch triggered as oxygen levels increase.

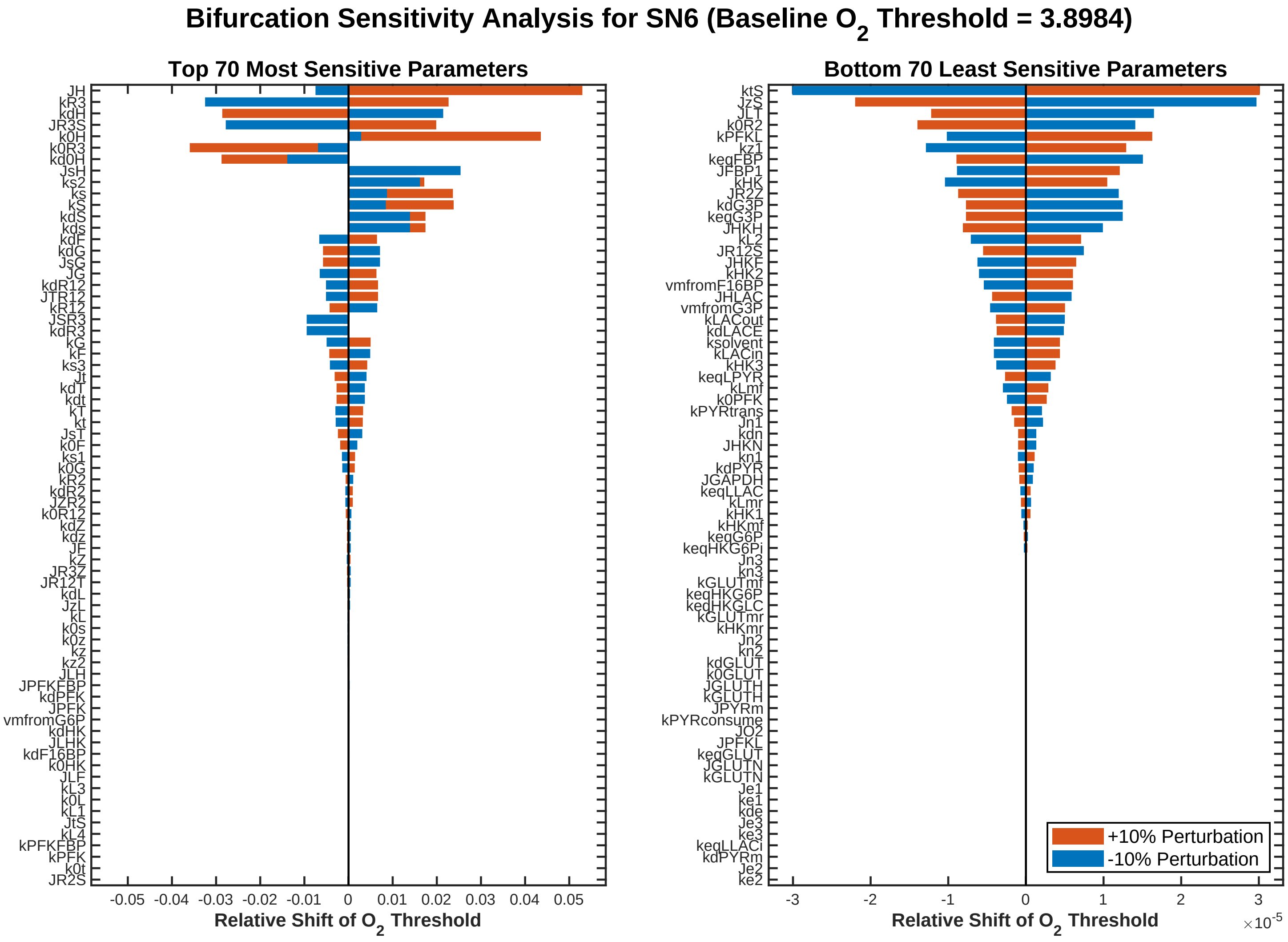

Figure S7: Parameter sensitivity analysis at the saddle-node bifurcation point SN6. This point corresponds to the initial EMT switch activated as oxygen levels begin to decrease.

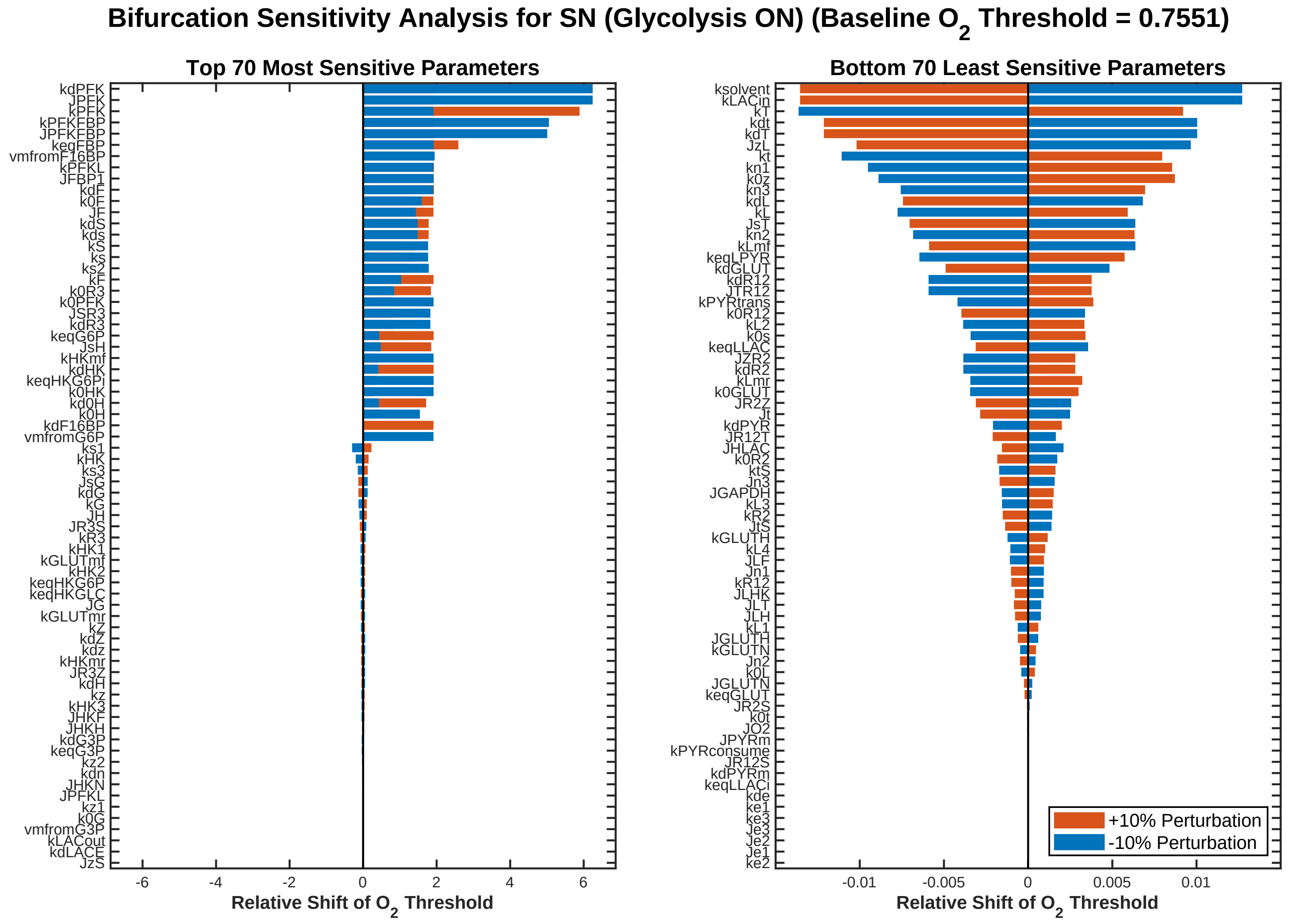

Figure S8: Parameter sensitivity analysis at the saddle-node bifurcation point triggering the glycolysis switch (Glycolysis ON) during hypoxia. This analysis highlights the parameters critical for the metabolic maintenance of the EMT state.

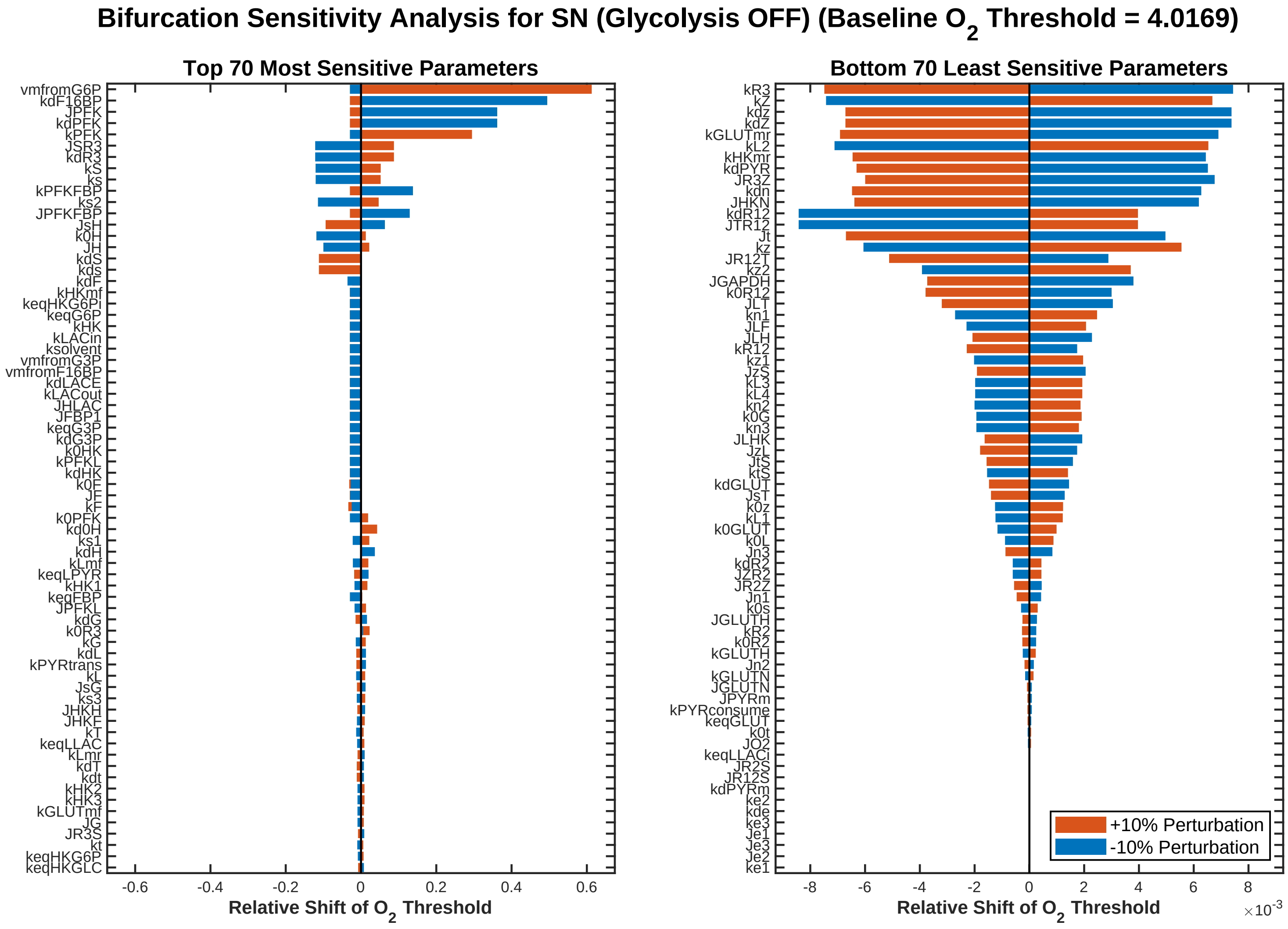

Figure S9: Parameter sensitivity analysis at the saddle-node bifurcation point deactivating the glycolysis switch (Glycolysis OFF) as oxygen levels recover.

Supplementary References

[15] Tian X J, Zhang H, Xing J. Coupled Reversible and Irreversible Bistable Switches Underlying TGFβ-Induced Epithelial to Mesenchymal Transition[J]. Biophysical Journal, 2013, 105(4): 1079-1089.

[17] Wang H Y, Zhang X P, Wang W. Regulation of Epithelial-to-Mesenchymal Transition in Hypoxia by the HIF-1α Network[J]. FEBS Letters, 2022, 596(3): 338-349.

[20] Tripathi S, Park J H, Pudakalakatti S, et al. A Mechanistic Modeling Framework Reveals the Key Principles Underlying Tumor Metabolism[J]. PLOS Computational Biology, 2022, 18(2): e1009841.

[69] Mulukutla B C, Yongky A, Daoutidis P, et al. Bistability in Glycolysis Pathway as a Physiological Switch in Energy Metabolism[J]. PLOS ONE, 2014, 9(6): e98756.

[82] Berra E, et al. Hypoxia-Inducible Factor-1α Escapes O2-Driven Proteasomal Degradation Irrespective of Its Subcellular Localization[J]. EMBO Reports, 2001, 2(7): 615-620.

[83] Moroz E, Carlin S, Dyomina K, et al. Real-Time Imaging of HIF-1α Stabilization and Degradation[J]. PLOS ONE, 2009, 4(4): e5077.

[84] Li F, Zhao H, Su M, et al. HnRNP-F Regulates EMT in Bladder Cancer by Mediating the Stabilization of Snail1 mRNA by Binding to Its 3′ UTR[J]. EBioMedicine, 2019, 45: 208-219.

[85] Dong L, Zhang X, Xiang W, et al. Post-Transcription Mediated Snail Stabilization Is Involved in Radiation Exposure-Induced Invasion and Migration of Hepatocarcinoma Cells[J]. Biomedicine & Pharmacotherapy, 2018, 103: 767-772.

[86] Zhu L, Xi P W, Li X X, et al. The RNA Binding Protein RBMS3 Inhibits the Metastasis of Breast Cancer by Regulating Twist1 Expression[J]. Journal of Experimental & Clinical Cancer Research, 2019, 38(1): 105.

[87] Xu R,Won J Y, Kim C H, et al. Roles of the Phosphorylation of Transcriptional Factors in Epithelial–Mesenchymal Transition[J]. Journal of Oncology, 2019, 2019: 5810465.

[88] Zhou B P, Deng J, Xia W, et al. Dual Regulation of Snail by GSK-3β-Mediated Phosphorylation in Control of Epithelial–Mesenchymal Transition[J]. Nature Cell Biology, 2004, 6(10): 931-940.

[89] Gantier M P, McCoy C E, Rusinova I, et al. Analysis of microRNA Turnover in Mammalian Cells Following Dicer1 Ablation[J]. Nucleic Acids Research, 2011, 39(13): 5692- 5703.

[90] Shieh H L, Chiang H L. In Vitro Reconstitution of Glucose-Induced Targeting of Fructose-1,6-Bisphosphatase into the Vacuole in Semi-Intact Yeast Cells[J]. Journal of Biological Chemistry, 1998, 273(6): 3381-3387.

[91] Regelmann J, Sch¨ule T, Josupeit F S, et al. Catabolite Degradation of Fructose-1,6-Bisphosphatase in the Yeast Saccharomyces cerevisiae: A Genome-Wide Screen Identifies Eight Novel GID Genes and Indicates the Existence of Two Degradation Pathways[J]. Molecular Biology of the Cell, 2003, 14(4): 1652-1663.

[92] Mansur N R, Meyer-Siegler K, Wurzer J C, et al. Cell Cycle Regulation of the Glyceraldehyde-3-Phosphate Dehydrogenase/Uracil DNA Glycosylase Gene in Normal Human Cells[J]. Nucleic Acids Research, 1993, 21(4): 993-998.

[93] Becker M, Newman S, Ismail-Beigi F. Stimulation of GLUT1 Glucose Transporter Expression in Response to Inhibition of Oxidative Phosphorylation: Role of Reduced Sulfhydryl Groups[J]. Molecular and Cellular Endocrinology, 1996, 121(2): 165-170.

[94] Camacho E T, Brager D, Elachouri G, et al. A Mathematical Analysis of Aerobic Glycolysis Triggered by Glucose Uptake in Cones[J]. Scientific Reports, 2019, 9(1): 4162.

[95] Ying M, Guo C, Hu X. The Quantitative Relationship between Isotopic and Net Contributions of Lactate and Glucose to the Tricarboxylic Acid (TCA) Cycle[J]. Journal of Biological Chemistry, 2019, 294(24): 9615-9630.
